# Maize Diterpenoid Sensing via the Ste3 A‐Pheromone Receptor Guide Oval Conidia of *Colletotrichum graminicola* to Host Roots

**DOI:** 10.1111/mpp.70155

**Published:** 2025-09-18

**Authors:** Anina Y. Rudolph, Carolin Schunke, Christoph Sasse, Luis Antelo, Jennifer Gerke, Gerhard H. Braus, Stefanie Pöggeler, Daniela E. Nordzieke

**Affiliations:** ^1^ Institute of Microbiology and Genetics, Genetics of Eukaryotic Microorganisms University of Göttingen, GZMB Göttingen Germany; ^2^ Institute of Microbiology and Genetics, Molecular Microbiology and Genetics University of Göttingen, GZMB Göttingen Germany; ^3^ Institute of Molecular Physiology, Microbiology and Biotechnology Johannes Gutenberg‐University of Mainz Mainz Germany; ^4^ Institute of Biotechnology and Drug Research gGmbH (IBWF) Mainz Germany

**Keywords:** chemotropic growth, *Colletotrichum graminicola*, diterpenoids, fungal GPCR, germination, oval conidia, root infection, *Zea mays*

## Abstract

*Colletotrichum graminicola*, the maize anthracnose fungus, is known for its ability to invade above‐ground tissues. This fungus forms two distinct asexual spore types in its life cycle, falcate conidia in acervuli on infected leaves and oval conidia in parenchyma cells in leaf and stem lesions. Our study reveals a previously unknown role for oval conidia in the infection of roots. We investigated whether root exudate from maize could redirect the growth of germlings generated by *C. graminicola* . Our results showed that only germlings derived from oval conidia were able to respond to root exudates, whereas those from falcate conidia did not. High‐performance liquid chromatography/mass spectrometry (HPLC–MS) analyses combined with biological assays and genetic studies identified diterpenoids from maize as attractants perceived by the a‐pheromone receptor CgSte3. We further explored the root–fungus interaction by analysing the germination of oval and falcate spores in soils of different composition. Oval conidia germinated rapidly under all conditions, whereas falcate conidia remained dormant and became highly vacuolated even in the presence of the host plant. Microscopic evaluation of root infection experiments showed that both conidia types attached to root material and formed penetration structures. However, only oval conidia enabled the pathogenic fungus to reach upper plant parts from infected roots. Our findings suggest a novel role for oval conidia in the infection of roots, highlighting the complexity of the anthracnose disease cycle.

## Graphical abstract

*Colletotrichum graminicola* produces two distinct asexual spore types: falcate conidia and oval conidia. Our study demonstrates that oval conidia possess adaptations that enable them to infect roots.

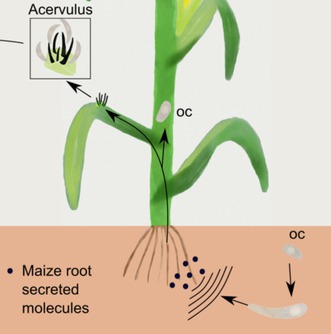

## 1 Introduction

The health of crop plants and fruits is affected by a variety of organisms, ranging from viruses and microorganisms (bacteria, oomycetes, fungi) to complex eukaryotes (nematodes, parasitic plants) (Strange and Scott 2005). Among these, fungi are prominent pathogens of major crops, reducing agricultural yield and quality by destroying plants preharvest and their fruits postharvest (Fisher et al. 2012; Silva et al. 2019). Timely, precise and sustainable crop protection strategies are essential, particularly as the burden of fungal plant diseases is expected to shift due to climate change (Chaloner et al. 2021).

The hemibiotrophic maize pathogen *Colletotrichum graminicola* is primarily known for infecting above‐ground tissues like leaves and stems, but can also invade the host roots (Bergstrom and Nicholson 1999). The known infection cycle includes the spread of the disease from primarily infected leaves or roots in a systemic infection, in which the hyphae colonise the fibres surrounding the vascular system and the vascular bundles themselves (Nordzieke, Sanken, et al. 2019; Sukno et al. 2008; Bergstrom and Nicholson 1999; Venard and Vaillancourt 2007; Belisário et al. 2022). With the ageing of the maize plant, this first predominantly symptomless systemic infection becomes visible by the formation of stalk rot (Bergstrom and Nicholson 1999). Current analyses estimate that anthracnose stalk rot (ASR) alone causes a 10%–20% annual loss in maize harvest worldwide (Belisário et al. 2022). *C. graminicola* produces two types of asexual spores: oval and falcate conidia. Both spore types are metabolically active and generated through different developmental processes in distinct plant tissues (Panaccione et al. 1989). Falcate conidia develop in acervuli on infected leaves, characterised by dark, spike‐like hyphae surrounded by masses of falcate spores. Oval conidia formation occurs in parenchyma cells in both leaf and stem lesions (Belisário et al. 2022; Sukno et al. 2008). They are generated from hyphae in a process that at first resembles branch formation. After an initial elongation, the oval conidia are released from the mother hyphae (Nordzieke, Sanken, et al. 2019). These differences influence the morphological and physiological properties of both spore types. Falcate conidia are single‐nucleate and dormant due to the production of mycosporines, while oval conidia contain variable nuclei and lack secondary metabolites promoting dormancy, leading to distinct infection strategies on leaves (Panaccione et al. 1989; Nordzieke, Sanken, et al. 2019; Nordzieke 2022): falcate conidia are highly efficient a penetration of the leaf surface as individuals. They form melanised appressoria from short germ tubes, which will subsequently form penetration pegs and infect the subjacent plant material. In contrast, oval conidia form penetration structures from hyphae, termed hyphopodia. These will only form when oval conidia are present in high spore densities. As a result, falcate conidia cause more severe symptoms on leaves when applied in lower spore numbers compared to oval conidia (Nordzieke, Sanken, et al. 2019). These findings suggest specific adaptation of falcate conidia for efficient leaf infection and raise the question of whether such specialisation exists also for other plant tissues.

Fungal plant pathogens specialise in entering host plants through distinct tissues (roots, shoots, stems, leaves) or at specific developmental stages (flower, fruit). Depending on the infected tissue, adapted signal‐sensing machineries and infection strategies are required. Foliar pathogens sense the plant surface via its physical and chemical properties, inducing germination and pathogenicity programmes (Kou and Naqvi 2016). In contrast, fungal root pathogens initiate infection before direct contact with the host, sensing nearby plants through root exudates that trigger germination and chemotropic growth towards plant‐secreted signals (Okubara and Paulitz 2005; Steinkellner et al. 2005; Narula et al. 2009; Vives‐Peris et al. 2020). Known chemical signals for this directed growth include sugars, amino acids, organic acids, fatty acids and plant‐derived enzymes as part of the plant defence response, guiding pathogens like *Fusarium oxysporum*, *Fusarium graminearum* and *Verticillium dahliae* to host roots, where they colonise root surfaces and invade the host (Deacon and Donaldson 1993; Donaldson and Deacon 1993; Ruttledge and Nelson 1997; Tyler 2002; Turrà et al. 2015; Sridhar et al. 2020; Vangalis et al. 2023).

In this study, we have analysed the potential of maize root exudate (MRE) on the attraction of the maize anthracnose fungus. Our analyses reveal that MRE attracts oval but not falcate conidia of *C. graminicola* . Chemical analysis and biological assays strongly indicate that maize root‐secreted diterpenoids act as attractants for chemotropic growth. We further identified the *C. graminicola* a‐pheromone receptor CgSte3 as the diterpenoid sensor in this host detection system. Furthermore, investigations of germination behaviour in soil and root infection experiments point to a probable better adaptation of oval conidia for the infection of maize roots compared to falcate conidia.

## 2 Results

### 2.1 Maize Root Exudate Elicit a Strong Chemotropic Response in *C. graminicola* Oval Conidia

Directed growth to plant‐secreted signals was described for several root‐infecting pathogenic fungi (Nordzieke, Fernandes, et al. 2019; Turrà et al. 2015; Sridhar et al. 2020). For the quantification of such a response, the chemotropic assay and its improvements are used in the community as standard approaches (Schunke et al. 2020; Turrà et al. 2015). Independent of the approach applied, fungal conidia are cultivated on or in a water agar matrix. At equal distances to both sides, signal compounds and solvent controls are applied in wells, generating gradients during the time of incubation. In the process of the experiment, conidia are allowed to sense the forming gradients. After a defined species‐dependent incubation time, microscopic observation is used to rate whether the germlings show a preferred growth direction towards the solvent control or the signal applied, which is expressed using the chemotropic index (< 0 repulsion by applied signal, 0 = no signal/no sensing of the signal, > 0 attraction by the signal).

**FIGURE 1:**
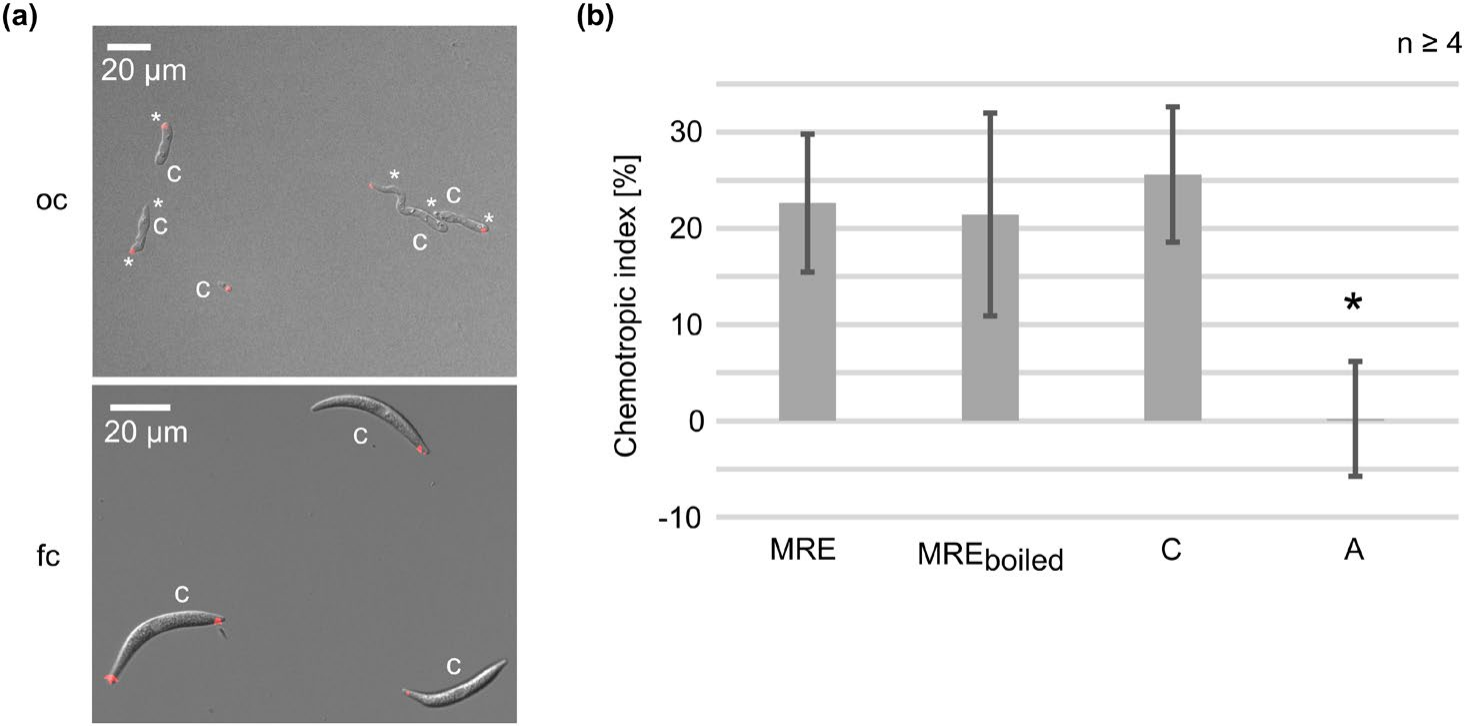
Chemotropic growth of *Colletotrichum graminicola* conidia to maize root exudate (MRE) and its fractions. (a) Oval (oc) and falcate conidia (fc) of *C. graminicola* expressing red‐labelled Arp1 (CgM2::Arp1‐TagRFP‐T, Groth et al. 2021) after 6 h of incubation facing a gradient of maize root exudate (MRE) in a 3D‐printed incubation chamber (Schunke et al. 2020), c = conidia, asterisks = germ tube, scale bar = 20 μm. (b) Chemotropic index of CgM2 oval conidia facing different gradients including MRE and boiled MRE. Chloroform (C) and aqueous (A) phases gained from extraction with chloroform/methanol of MRE. Error bars represent SD calculated from *n* ≥ 4 experiments, *p < 0.05.

In our laboratory, we use a 3D‐printed device as an improved tool for the original assay. Due to its composition, this tool enables easy assembly of a reaction chamber, standardises the application of gradients and allows the observation of chemotropically growing germlings with the help of fluorescent markers (Schunke et al. 2020). In a first experiment applying this 3D‐printed device, we aimed to see whether oval or falcate conidia were able to sense MRE gradients and to direct their growth towards those substances. To visualise the sensing of the MRE gradient, a wild‐type strain expressing the polarity marker CgArp1‐TagRFP‐T was used. This polarity marker labels the current side of polar growth of fungal hyphae, providing information about the part of a fungal germling involved in growth redirection (Groth et al. 2021). After 6 h of incubation, oval conidia are well germinated. They showed typical localisation of the polarity marker CgArp1‐TagRFP‐T to the subapical region of growing hyphae, whereas it was absent from the non‐growing parts of the germlings. The corresponding hyphal tips pointed mainly to the gradient of MRE, indicating active hyphal growth of the germlings to the root‐secreted signals (Figure 1a,b). In contrast, we were unable to detect any germinated falcate conidia, indicating that those spores are unable to germinate and redirect growth under the experimental conditions and timing (Figure 1a). Additionally, the red‐fluorescent CgArp1‐TagRFP‐T marker showed no clear localisation towards MRE or the applied solvent control, indicating that no sensing of the applied molecules takes place. To exclude that falcate conidia might require increased incubation times, we tested different time points up to 48 h, including the re‐establishment of MRE gradients. However, we were never able to see any germination of falcate conidia.

### 2.2 The CgSte3 A‐Pheromone Receptor Is Responsible for Diterpenoid Sensing in *C. graminicola*

Next, we sought to identify the attracting molecule incorporated in MRE. In plant–fungus interaction studies with other phytopathogenic species, secreted peroxidases from the host were identified as chemoattractant signals (Turrà et al. 2015; Sridhar et al. 2020; Vangalis et al. 2023). Because the enzymatic activity of those enzymes is required for fungal attraction, boiling of tomato root exudate abolishes its attracting potential (Turrà et al. 2015; Nordzieke, Fernandes, et al. 2019). In contrast, boiling of MRE did not diminish the chemotropic growth of *C. graminicola* germlings (Figure 1b), indicating that peroxidases are not the attractant molecule secreted from maize roots. Chloroform/methanol extractions were performed to explore further chemical properties of the active MRE, resulting in two phases with different chemical properties. The chloroform phase, incorporating unpolar compounds such as lipids or lipid‐associated molecules, induced a strong chemotropic response after solvent evaporation and resuspension in water. No directed growth was induced by the water phase, which typically contains polar substances (Figure 1b).

**FIGURE 2:**
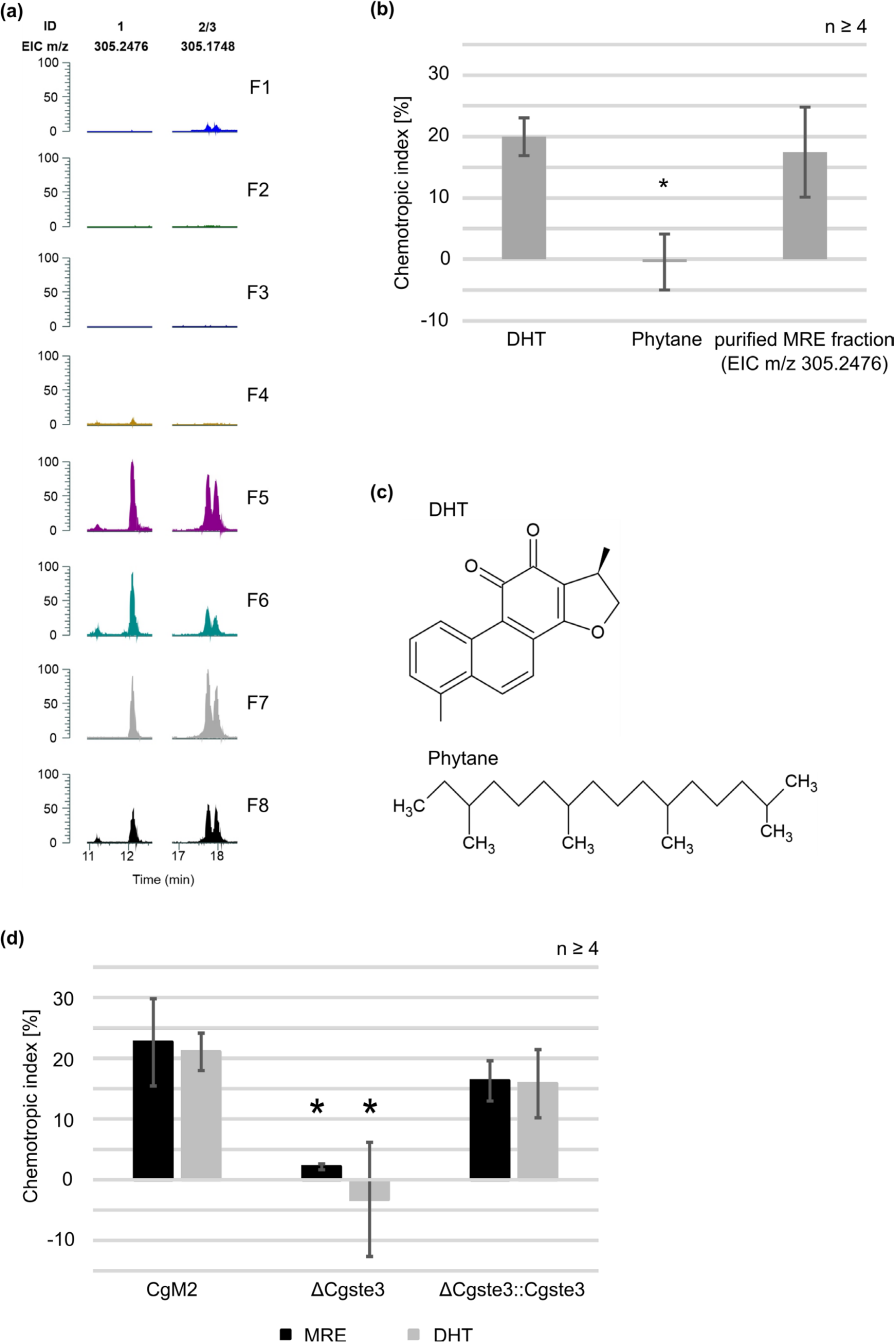
Diterpenoids from maize root exudate (MRE) and other sources are perceived by the a‐pheromone receptor CgSte3 of *Colletotrichum graminicola* . (a) Extracted ion chromatograms (EICs) from HPLC/MS analyses of MRE factions attracting (F1–F4) or not‐attracting (F5–F8) extracted using ethyl acetate (1:1) or chloroform:methanol (1.8:2.2): F1 = demineralised water extracted with ethyl acetate; F2 = demineralised water extracted with chloroform/methanol, chloroform phase; F3 = demineralised water extracted with chloroform/methanol, aqueous phase; F4 = MRE extracted with chloroform/methanol, aqueous phase; F5 = MRE extracted with ethyl acetate; F6 = boiled MRE extracted with ethyl acetate; F7 = MRE extracted with chloroform/methanol, chloroform phase; F8 = MRE obtained from *C. graminicola* root‐infected plants extracted with ethyl acetate. ID = number of the compound in this study, the identified metabolites 1 und 2/3 were identified in *n* = 3 replicates. (b) Chemotropic growth of *C. graminicola* germlings derived from oval conidia towards diterpenoids (dihydrotanshinone I (DHT I), phytane) and purified MRE fraction containing the metabolite with EIC *m*/*z* 305.2476. Error bars represent SD calculated from *n* ≥ 4 experiments, *p < 0.05, calculated with two‐tailed *t* test (c) Chemical structure of the tricyclic diterpenoid dihydrotanshinone I (DHT) and phytane, a linear diterpenoid. Chemical structures drawn with ACD/ChemSketch. (d) Chemotropic index of CgM2, ΔCgste3 and ΔCgste3::Cgste3 germlings derived from oval conidia facing gradients of different chemoattractants (MRE, DHT). Statistical significance was calculated with CgM2 as reference. Error bars represent SD calculated from *n* ≥ 4 experiments, *p < 0.05, calculated with two‐tailed *t* test.

For the identification of the attracting molecules in MRE, attracting and not‐attracting MRE fractions were extracted using acetonitrile and analysed using HPLC/MS analyses. To test whether previous biotic stress might change the chemical composition of the MRE, exudates generated from previously root‐infected plants were also included. Three molecules in the active fractions were consistently detected in three independent experiments, at the same time absent from the negative controls (Figure 2a, Figure S1). Interestingly, the double peak eluting at approximately 18 min was absent in the third replicate, except for the MRE sample from *C. graminicola* root‐infected plants. This indicates a flexibility of MRE composition dependent on the defence status of the plant. Comparing UV and MS2 spectra of the chemical compound released at 12 min (mass: 305.2476) with known diterpenoids from maize, we found the colourless 3β,15,16‐trihydroxydolabrene (trihydroxydolabrene, THD) having a very similar MS2 spectrum (Figure S2) (Mafu et al. 2018). After a first separation using HPLC, the obtained fractions were tested regarding their potential to attract *C. graminicola* oval conidia (Figure S3). Fractions 4–6 and 12–13 were chosen for further purification and testing. Using preparative HPLC, we were able to purify the peak corresponding to the mass of 305.2476 and verified its attraction potential in a chemotropic assay (Figure 2b). We conclude it is very likely that the maize root‐derived diterpenoid THD is attracting *C. graminicola* oval conidia to its host. In order to test non‐maize diterpenoids for their attraction potential, we chose dihydrotanshinone I (DHT, originated from *Salvia* species), a cyclic diterpenoid like THD and phytane, a linear diterpenoid. As depicted in Figure 2b,c, oval conidia of *C. graminicola* are attracted by DHT, but not phytane.

In different *Fusarium* species, the pheromone receptors Ste2 (α‐pheromone receptor) and Ste3 (a‐pheromone receptor) activate the downstream cell wall integrity (CWI) MAPK cascade, resulting in the redirection of germling growth towards plant‐derived peroxidases (Turrà et al. 2015; Sridhar et al. 2020; Sharma et al. 2022; Ramaswe et al. 2024). In the *Colletotrichum* genus, several species have lost one or the other pheromone receptors and the corresponding pheromones. For example, in *Colletotrichum higginsianum*, both receptor and pheromone pairs are present. In *Colletotrichum tofieldiae*, the Ste2 receptor is missing, but the a‐pheromone is still encoded. In *C. graminicola* , the fungus investigated in this study, CgSte2 and the α‐pheromone are not encoded in the genome, but CgSte3 receptor and the a‐pheromone are present (Wilson et al. 2021).

A protein homology search (Blastp) based on the *F. oxysporum* FoSte3 amino acid sequence was used to identify GLRG_03765 homologue in *C. graminicola* . AlphaFold protein structure prediction revealed a typical structure for seven transmembrane receptor proteins, with a C‐terminal extension (Figure S4). Alignments with other Ste3 protein sequences of *Saccharomyces cerevisiae* , *F*. *oxysporum* and *F. graminearum* show a high sequence similarity, although CgSte3's C‐terminus is significantly shorter compared to the C‐termini of the other fungi. Based on this first analysis, a *Cgste3* deletion mutant and complementing strain were generated and verified (Figures S5 and S6). Intriguingly, germlings derived from ΔCgste3 oval conidia were unable to redirect their growth to MRE and DHT (Figure 2d), providing evidence that the a‐pheromone receptor CgSte3 is required for the sensing of diterpenoid plant signals by *C. graminicola* .

### 2.3 Successful Germination of *C. graminicola* Conidia in Soil Determines the Ability to Infect Maize Roots

Besides the ability to redirect growth to MRE, one of the most prominent differences between oval and falcate conidia is the specific germination patterns. Germination of falcate conidia is regulated by the secondary metabolites mycosporines, serving as self‐germination inhibitors in high population densities and in the absence of monosaccharides (Leite and Nicholson 1992). In contrast, oval conidia do not secrete mycosporine‐derivatives and thus germinate rapidly even when nutrients are absent (Nordzieke, Sanken, et al. 2019). These spore‐type specific adaptations suggest that there might be divergent germination patterns in soil, which influence the abilities of both spore types to infect the host plant's roots.

**FIGURE 3:**
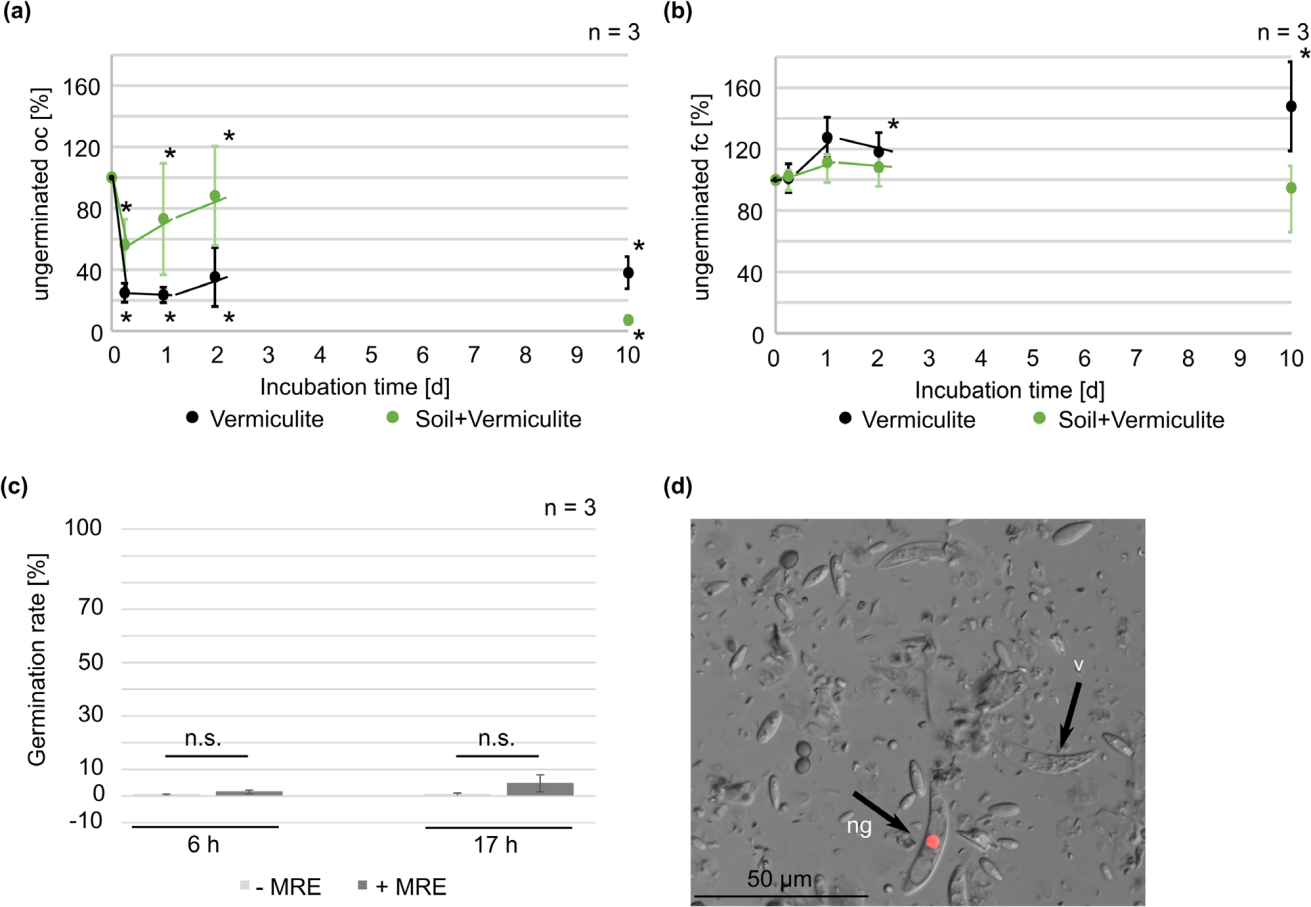
Germination of *Colletotrichum graminicola* conidia. Germination of oval (oc, (a)) and falcate (fc, (b)) conidia in vermiculite and a mixture of soil and vermiculite (4 + 1). Quantification of ungerminated conidia after incubation for 0 h, 6 h, 24 h, 48 h, 10 day in a Neubauer counting chamber, *n* = 3 experiments, *p < 0.05, calculated with two‐tailed *t* test compared to timepoint 0 h. (c) Germination of falcate conidia on water agar after 6 h and 17 h with and without the supplement of maize root exudate (MRE), *n* = 3 replicates, the two‐tailed *t* test ws performed. *p < 0.05, n. s. = not significant. (d) Falcate conidia (black arrows) in soil after a 10 day co‐incubation with maize roots in vermiculite, ng = not germinated, v = vacuolated, scale bar = 50 μm.

The germination ability of oval and falcate conidia in soil of different compositions was analysed to address this hypothesis (Figure 3a,b, Figure S7). Because in pre‐experiments, we realised that once germinated, fungal mycelium will stick to soil particles, making adequate quantifications of conidial germination impossible, we chose another approach: after incubation, the spore‐contaminated soil was washed with a defined amount of water. The number of ungerminated spores washed out was determined. Independent of the substrate used, the amount of ungerminated oval conidia dropped down within 6 h of incubation (vermiculite: 75% reduction; vermiculite soil mix: 43.8% reduction). Surprisingly, after the first decrease in oval conidia numbers, their amount increased again within the first 2 days of inoculation in both substrates. We speculate that this intermediate increase could be due to new oval conidia produced in the watery substrate. After 10 days, the main fractions of conidia were germinated in both spore samples (vermiculite: 62% reduction; vermiculite soil mix: 92.8% reduction). In contrast, for falcate conidia the amount of ungerminated spores detected remained stable, also after prolonged incubation for 10 day and other soil substrates tested.

It has been shown for several root‐interacting fungi that root exudate can induce germination (Steinkellner et al. 2005, Li et al. 2013, Eze 2010). We thus tested whether MRE is able to induce germination of falcate conidia on axenic culture or in soil. For the first experiments, defined drops of falcate conidia were incubated on water agar with or without the addition of MRE. As depicted in Figure 3c, MRE was unable to induce germination in falcate conidia. We further analysed the effect of living maize plants on the germination ability. Consistent with the previous results, only ungerminated falcate conidia were detectable after 10 days of plant‐co‐incubation (Figure 3d). Furthermore, many highly vacuolated and dead falcate conidia were detected, indicating that this spore type is unable to survive for a longer time in soil (Figure 3d).

### 2.4 Root Dipping in *C. graminicola* Oval Conidia Spore Suspension Induces a Symptomless Infection and Disease Spread Into Maize Stems

**FIGURE 4:**
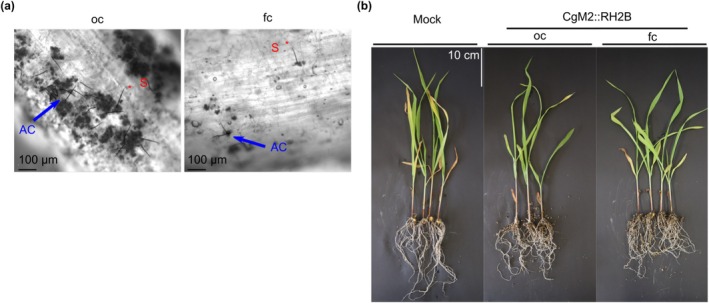
Symptom development after root infection of *Zea mays* with *Colletotrichum graminicola* . (a–c) Roots of 5‐day‐old plants were dipped in oval (oc) or falcate (fc) conidial suspensions of the strain CgM2::RH2B allowing for the detection of red‐fluorescent nuclei during microscopic visualisation. (a) *C. graminicola* development on roots 4 days after root dipping, AC = acervulus (blue arrow), S = seta (red asterisk), scale bar = 100 μm. (b) Representative symptom development 21 days after root dipping in conidial suspensions and cultivation in vermiculite, scale bar = 10 cm.

To test whether the observed differences of oval and falcate conidia regarding MRE sensing and germination patterns in soil might affect the ability of the spore types to infect roots, a *C. graminicola* wild‐type strain expressing a tdTomato‐labelled histone marker, CgM2::RH2B (Nordzieke, Sanken, et al. 2019), was used for root infection experiments. CgM2::RH2B oval or falcate conidia suspensions were used as inoculum for the infection of 5‐day‐old maize roots prior to incubation under humid conditions. The microscopic evaluation showed production of several fungal structures as previously observed for root infection with falcate conidia (Sukno et al. 2008, Figure S8). At 4 days post‐infection, production of acervuli, the asexual fruiting bodies giving rise to falcate conidia, was observed for both conidia types (Figure 4a). Additionally, development of typical root colonisation structures like long, fast‐growing runner hyphae, hyphopodia for root penetration and dark‐pigmented resting structures (microsclerotia) were observed upon oval and falcate conidia colonialisation (Figure S8).

To analyse symptom development on the growing plant, 5‐day‐old plants dipped in conidial suspensions were planted out in vermiculite. After 21 days of growth under humid conditions, the length and biomass of above‐ground tissue was measured. As depicted in Figure 4b and Figure S9, no significant differences to mock inoculation were observed. A real‐time PCR approach was used to test for possible symptomless systemic infection by using gDNA extracted from surface‐sterilised stem samples as a template. Specific detection of *C. graminicola* was ensured by the design of oligonucleotides binding in the species‐specific region of rDNA internal transcribed spacers ITS1 and ITS2 (Figure S10). In total, we tested eight stems of mock, oval conidia and falcate conidia root infections. In the material of four stems derived from oval conidia‐infected plants, fungal biomass was detected (Table S4). In contrast, *C. graminicola* gDNA was absent in plants treated with falcate conidia and mock samples. Together, these results indicate successful disease spreading in planta only following oval conidia infection.

## 3 Discussion

**FIGURE 5:**
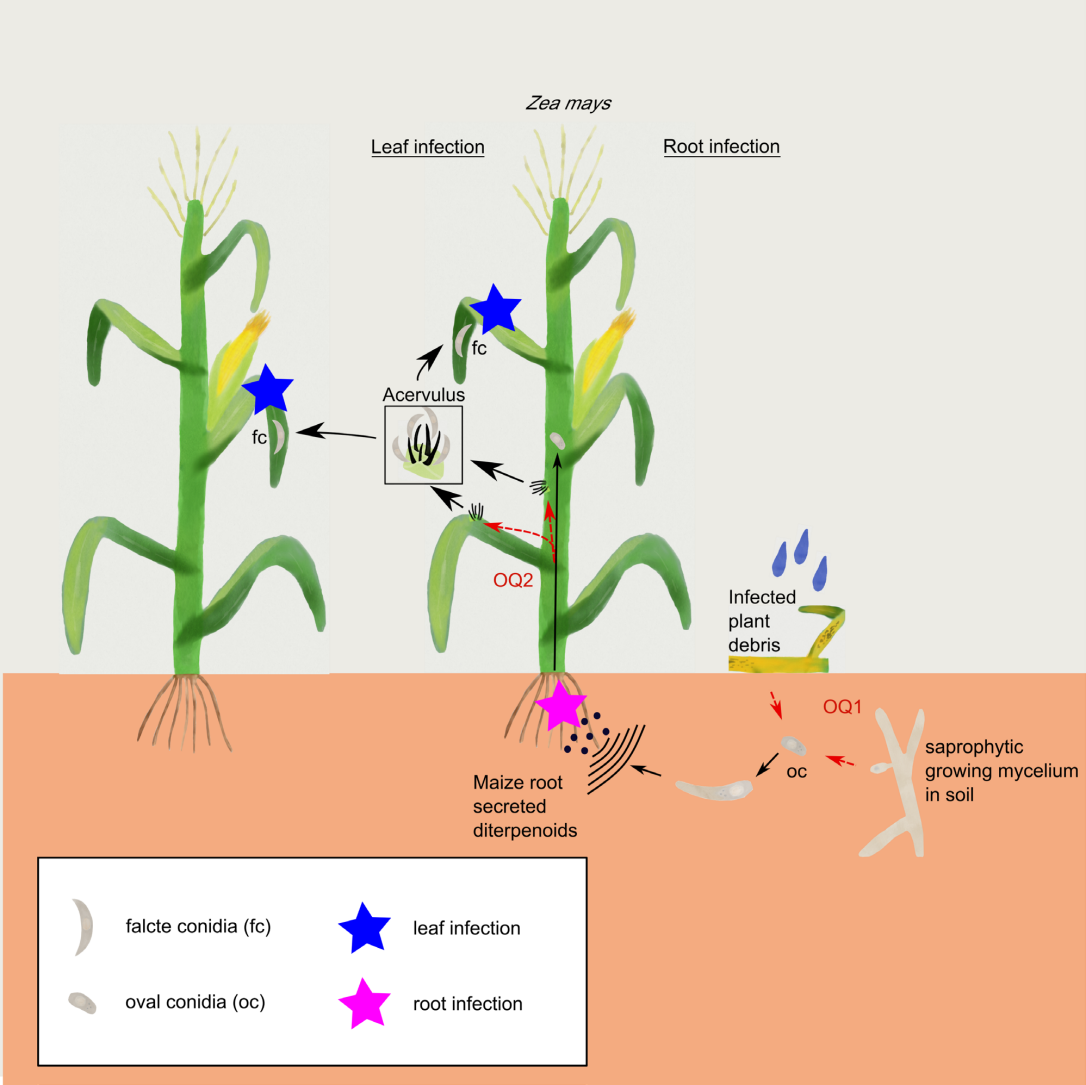
Working model of a putative extended anthracnose infection cycle. Oval conidia (oc) germinate in soil and redirect their growth to diterpenoids secreted from maize roots. Upon reaching host roots, infection starts followed by spreading of the fungus into plant stem material in a symptomless infection. When senescence occurs or the fungus reaches nodules plates, anthracnose stalk rot (ASR) or anthracnose leaf blight (ALB) occurs, resulting in the formation of falcate conidia (fc) in acervuli on plant surfaces. From these, falcate conidia are washed out from acervuli by wind and rain, thereby reaching other plant material of the same plant or neighbouring plant, resulting in fast above‐ground disease spread. Open questions are indicated in red text and dashed red arrows: (OQ1) How do oval conidia end up in the soil in the first place? In previous publications, oval conidia were retrieved from infected plant material. In this study, we also found indications that oval conidia could be produced directly in soil. However, both hypotheses have to be verified in future studies. (OQ2) How do oval conidia contribute to the formation of ASR and ALB? To answer this question, long‐term experiments with adult maize plants are required. Information for the model are a summary of the current study and previous publications (Nordzieke, Sanken, et al. 2019; Bergstrom and Nicholson 1999; Venard et al. 2008; Venard and Vaillancourt 2007; Chaky et al. 2001; Belisário et al. 2022; Panaccione et al. 1989).

The main finding of this study is that oval conidia efficiently sense diterpenoids as parts of the exudate secreted from maize roots, providing growth orientation towards the host. In detailed germination and root infection experiments, we show that oval conidia do germinate fast in soil and spread from primary infected roots into stem tissues. In contrast, falcate conidia cannot germinate in soil, do not respond to root exudate treatments and are unable to spread from plant roots into the shoot. Together, these results demonstrate that oval conidia show several characteristics of plant root‐pathogenic fungi, whereas falcate conidia do not. These findings point to a probable undiscovered branch of the anthracnose disease cycle, with important implications for future crop protection strategies (Figure 5).

Exudates from plant roots play several critical roles in plant health, such as adapting soil pH to enhance nutrient uptake, chelating toxic compounds, attracting beneficial microorganisms and releasing antimicrobial substances to defend against pathogens (Vives‐Peris et al. 2020; Zhang et al. 2020). During pathogen attacks, plants further adapt the composition of root exudates by synthesising new antimicrobial compounds (Lanoue et al. 2010). Prominent examples of such antimicrobial compounds include class III peroxidases, which are secreted from plant roots and participate in reactive oxygen species defence pathways (Mika et al. 2010; Bindschedler et al. 2006; Wen et al. 2007). However, several pathogenic fungi can sense these peroxidases and, rather than being harmed or repelled, use them to guide themselves towards nearby host plants through a sophisticated amplification and sensing system involving fungal NADPH oxidase and pheromone receptors (Nordzieke, Fernandes, et al. 2019; Turrà et al. 2015; Sridhar et al. 2020; Vangalis et al. 2023). In this study, we describe for the first time the ability of *C. graminicola* oval conidia to sense maize root exudates, provoking a robust chemotropic response. Chemical analysis and fractionation experiments revealed that the active substances secreted from maize roots are heat‐stable and soluble in chloroform (Figure 1). Comparisons of the masses obtained from HPLC/MS analysis with known secondary metabolites showed a high similarity with diterpenoids. UV and MS2 spectra of one compound matched with 3β,15,16‐trihydroxydolabrene (THD). This colourless diterpenoid, secreted from maize roots, has antimicrobial properties (Mafu et al. 2018). Diterpenoids are involved in terpenoid‐based defences against various maize pathogens, including fungi such as *Rhizopus microsporus*, *Ustilago maydis* and *F. graminearum*, as well as herbivorous and insect species (Zhou et al. 2024). Similar to class III peroxidases, the biosynthesis of several diterpenoids is induced by plant pathogens and defence‐related phytohormones such as jasmonic acid and ethylene (Schmelz et al. 2011; Hiraga et al. 2001; Kidwai et al. 2020; Huffaker et al. 2011). Our results show that diterpenoid and maize root exudate (MRE) perception in *C. graminicola* is mediated by the α‐pheromone receptor Ste3, indicating a certain level of conservation in the sensing of host‐secreted molecules despite the different chemical properties of the attractants (Turrà et al. 2015; Sridhar et al. 2020; Vangalis et al. 2023). However, unlike *Fusarium* and *Verticillium* species, the absence of the α‐pheromone receptor Ste2 (Wilson et al. 2021) does not impede the perception of maize root‐secreted signals, suggesting differences in the perception process despite their similarities in the different fungal species. These results indicate that the ability to use plant defence molecules for host detection has evolved multiple times and can adapt to different chemical compounds depending on the fungal–plant pathosystem involved.

This study emphasises the importance of proper germination for plant infection. We assume that the reasons for the incapability of falcate conidia to germinate in soil are mainly based on the tight regulation of spore dormancy, for example by the generation and secretion of mycosporines. These secondary metabolites are germination inhibitors, which are produced at high spore densities, low availability of monosaccharides like glucose, and in the absence of a living plant surface (Leite and Nicholson 1992; Nordzieke, Sanken, et al. 2019). Dependent on the stimulus, falcate conidia will induce vegetative growth (monosaccharide sensing) or start the formation of appressoria for plant penetration (surface recognition). The effect of mycosporines is typical for a microbial quorum‐sensing (QS) molecule: QS molecules are constantly secreted and accumulate in the surrounding area of the secretion spot. Once a certain concentration threshold is reached, gene expression changes and a switch in growth and general activity of the population takes place, which is triggered relative to its density (Tian et al. 2021). Earlier work showed that mycosporines can be washed off falcate spores (Leite and Nicholson 1992); however, in our hands, seven repetitions of rigorous washing were required to see any effect on germination, making it unlikely that such is relevant in nature. In the soil, monosaccharides, which could repress mycosporine biosynthesis, are mainly present due to plant exudates (Gunina and Kuzyakov 2015). Our results demonstrate that in axenic cultures, and applying MRE directly on falcate conidia, a minimal and not statistically relevant induction of germination takes place. However, in natural soil conditions, exudates from the host are insufficient to inhibit mycosporine generation and break the dormancy of falcate conidia (Figure 3). Although mycosporines were identified as germination regulators of *C. graminicola* in the 1990s (Leite and Nicholson 1992), the pathways governing their biosynthesis and regulation remain largely unknown, necessitating further investigation to clarify this aspect of the maize anthracnose pathogen.

In conclusion, our results indicate that oval conidia show several characteristics of a root‐pathogenic fungus: oval conidia germinate fast in soil and sense molecules exuded from roots of its host. After infection of roots by oval conidia, the fungus spreads successfully into maize stems. However, it is not clear how these spores can enter the soil, although we found indications that those spores could be generated in wet soil. Together with previous reports about a highly adapted behaviour of falcate conidia for leaf infection, our findings support a new view of the overall anthracnose disease cycle, in which two different spore types have evolved to infect distinct plant tissues. Further investigations are required to show the relevance of the differences seen also for the disease implementation in nature.

## 4 Experimental Procedures

### 4.1 Strains, Growth Conditions and Collection of Spores

The sequenced *C. graminicola* (teleomorph *Glomerella graminicola* (Forgey et al. 1978)) wild‐type strain M2 (also referred to as M1.001) was provided by H. Deising (University of Halle, Germany) and used in this study (O'Connell et al. 2012). Falcate conidia production of *C. graminicola* was induced by growth on oat meal agar (OMA; 1 L contains 50 g ground oat flakes (Alnatura, Bioland)) and the exposure to red (660–665 nm) and blue (450–455 nm) light in a ratio of 3:1 at 23°C (Nordzieke, Sanken, et al. 2019). After 14–21 day, falcate conidia were collected with a 0.02% Tween 20 solution, centrifuged and resuspended in a 0.01% Tween 20 solution to enable proper determination of spore concentrations. For the generation of oval conidia, liquid complex medium containing sucrose (CMS, 1% glucose, 0.1% yeast extract, 0.1% peptone, 0.5 M sucrose, 5.3 mM Ca(NO_3_) (2) 0.073 mM KH_2_PO_4_, 1.04 mM MgSO_4_, 0.46 mM NaCl) was inoculated with 50 μL falcate spore stock solution or mycelial plugs. After germination induction at 23°C for 2–3 days (with agitation at 80 rpm), further incubation took place for 5–8 day without agitation in darkness. Oval conidia were collected by filtration through Miracloth (Merck‐Millipore) followed by centrifugation and a single washing step.

### 4.2 Generation of Maize Root Exudate

Roots of four 9‐day‐old maize plants grown in vermiculite (Vermiculite Palabora, grain size 2–3 mm, Isola Vermiculite GmbH) were rinsed and placed in 40 mL sterile demineralised water. After incubation for a further 5 day in a plant chamber using standard plant growth conditions, the plants were discarded, the MRE was sterilised with a 0.45 μm filter (86.1197, Sarstedt), and frozen in aliquots at −20°C. For the generation of exudate from stressed plant roots, plants were first dipped in spore solutions (see experimental procedure described below) before the incubation in 40 mL sterile demineralised water took place for 5 day.

### 4.3 Chemical Analysis of Maize Root Exudate

For the identification of probable bioactive substances in MRE, HPLC‐MS analyses were performed with MRE extracts generated using ethyl acetate (1:1) or chloroform:methanol (1.8:2:2). Prior to HPLC‐MS analysis, the extracts were dissolved in 500 μL methanol. The resulting suspensions were centrifuged at 15,700 *g* for 10 min at 4°C to remove undissolved particles. 400 μL of the supernatants were transferred to LC‐MS glass vials. A Q‐Exactive Focus orbitrap mass spectrometer coupled to a Dionex Ultimate 3000 HPLC (Thermo Fisher Scientific) was used for analysis. HPLC was carried out using a Acclaim 120, C18 column (5 μm, 120 Å, 4.6 × 100 mm). 5 μL of each sample was injected for analysis. The mobile phase consisted of a linear gradient from 5% to 95% (vol/vol) acetonitrile/0.1% formic acid over 20 min, followed by 10 min at 95% (vol/vol) acetonitrile/0.1% formic acid with a flow rate of 0.8 mL/min at 30°C. The measurements were performed in positive and negative modes with a mass range of 70–1050 *m*/*z*. UV/vis spectra were recorded with a Thermo Scientific Dionex UltiMate 3000 Diode Array Detector. MS2 data were recorded at a HCD energy of 35 eV. For data analysis the software FreeStyleTM v. 1.4 (Thermo Fisher Scientific) and the database ‘Dictionary of Natural Products’ were used.

For the identification of active MRE fractions, 8.5 L of MRE was extracted with equal amounts of ethyl acetate. After solvent evaporation in a rotary evaporator, the resulting extract (387 mg) was applied onto a preparative HPLC system. HPLC purification was performed on a Shimadzu LC system equipped with a LC‐20AP pump, an FCV‐200AL valve, a CBM‐40 controller and an FRC‐10A fraction collector. Separations were done on a preparative reversed phase C18 column (Waters Sunfire C18, particle size 5 μm, 19 × 250 mm) and a semipreparative reversed phase C18 column (Waters Sunfire C18, particle size 5 μm, 10 × 250 mm) at room temperature. A linear gradient starting from 99% of 0.1% trifluoroacetic acid and 1% acetonitrile to 100% acetonitrile in 20 min, then maintaining 100% acetonitrile for 3 min and an additional equilibration time of 7 min was used at a flow rate of 17.1 mL/min. The injection volume of the sample solution was 1 mL. The volume of collected fractions was 3 mL. A Shimadzu SPD‐M40A diode array detector was used from 200 to 800 nm for recording of spectra and detection of separated secondary metabolites at 210, 250, 300, 350 and 400 nm. Data and spectra were analysed using the software LabSolution 5.110. LC‐ESI‐MS analysis was performed on a Shimadzu LC system equipped with two LC‐40D pumps, a DGU‐405 degassing unit, a SIL 40C autosampler, a SCL‐40 controller and a CTO‐40C column oven. Mass analysis was performed with a single quadrupole LCMS‐2020 system. Separations were done on an analytical reversed phase C18 column (Macherey Nagel, Nucleoshell RP18, particle size 2.7 μm, 2 × 100 mm) at 40°C. A gradient starting from 95% of 0.1% formic acid and 5% acetonitrile with a holding time of 2 min to 100% acetonitrile in 6 min, then maintaining 100% acetonitrile for 2 min and an additional equilibration time of 4 min was used at a flow rate of 0.3 mL/min. The injection volume of the sample solution was 1 μL. A Shimadzu SPD‐M40A diode array detector was used from 200 to 800 nm for recording of spectra and detection of secondary metabolites at 210 nm, 250 nm, 300 nm, 350 nm, 400 nm, 450 nm and 500 nm. A single quadrupole LCMS System‐2020 (Shimadzu) with ESI was used with the following parameters: Scan mode, positive and negative, starting from 150 to 1000 *m*/*z* with acquisition time from 2 to 10 min. DL temperature 250°C, nebulising gas flow 1.5 L/min, drying gas flow 10 L/min and the temperature of the heat block was 200°C. Data and spectra were analysed using the software LabSolution v. 5.109.

### 4.4 Chemotropic Assay

Chemotropism is defined as directed growth along a gradient of a given chemical stimulus. This growth can be positive (towards a chemical attractant) or negative (away from chemical repellant). The first description of a methodical approach to quantitatively access chemotropic growth response in filamentous fungi was provided by Turrà et al. (2015). To display positive and negative chemotropic growth responses in an easily approachable way, they introduced the ‘chemotropic index’, which indicates the difference in a preferred growth direction compared to a situation in which neither attraction nor repulsion occurs (Turrà et al. 2015). Although the usage of the ‘chemotropic index’ has some drawbacks (Schunke et al. 2020), it is, currently, the standard depiction used in research of chemically induced chemotropic growth in fungi (Turrà et al. 2015; Rudolph et al. 2024; Nordzieke, Fernandes, et al. 2019; Sharma et al. 2022; Vangalis et al. 2023; Vitale et al. 2019; Lombardi et al. 2018).

To improve the original method, we have generated a 3D‐printed frame, fitting to microscopic slides. By pouring water agar into the frame, wells for the application of chemotropic stimuli and solvent controls are formed, enabling the formation of a signal gradient. Conidia are applied (20 μL of a spore solution with c = 5*10^5^ mL^−1^) between both wells. After an incubation time of 6 h, the growth direction of germlings at a defined distance to the wells, and thus the forming signalling gradient, is analysed (Schunke et al. 2020). Another methodical advancement is the use of strains expressing the Arp1‐TagRFP‐T polarity marker, which indicates the current axis of polar growth and is absent from hyphae that are not growing. Thus, this marker can be used to improve the readout of the chemotropic growth assay, in which only actively growing, and thus sensing, hyphae are included in the evaluation. Furthermore, Arp1‐TagRFP‐T accumulates in ungerminated conidia at the site of future germination, enabling those conidia to be included in the evaluation (Groth et al. 2021). In this study, quantification of chemotropic growth of different *C. graminicola* wild‐type (CgM2) and mutant strains derived from oval or falcate conidia was assessed towards MRE, different MRE fractions, DHT (Sigma‐Aldrich) and phytane (Sigma‐Aldrich) using the described 3D‐printed device (Schunke et al. 2020). DHT and phytane were first dissolved in a 13% ethanol solution and subsequently diluted with distilled water until the final concentration of 128 μM. This concentration was previously used to study peroxidase‐mediated attraction of other fungi to their host plants (Turrà et al. 2015; Nordzieke, Fernandes, et al. 2019). To improve the readout, CgM2 strains expressing the polarity marker Arp1‐TagRFP‐T were used to analyse the reaction of oval and falcate spores to MRE exposure (Figure 1, Groth et al. 2021). For each biological replicate, the growth direction of at least 50 germlings was determined.

### 4.5 Identification of the Pheromone A‐Factor Receptor Homologue in *C. graminicola*

The *C. graminicola* gene encoding the predicted pheromone a‐factor receptor CgSte3 (GLRG_03765) was identified by sequence similarity searches against the orthologue of *F. oxysporum* f. sp. *lycopersici* protein (FOXG_02147) using BLASTp (FungiDB; https://fungidb.org). Structure prediction with AlphaFold Protein Structure Database (https://alphafold.ebi.ac.uk/) showed the typical structure of a G‐protein coupled receptor with six transmembrane domains and a long C‐terminal extension (Figure S4). Protein alignment was performed with ClustalOmega (https://www.ebi.ac.uk/jdispatcher/msa/clustalo; alignment style: Nightingale, colour scheme: clustal2) with sequences of homologous proteins of *Saccharomyces cerevisiae* (YKL178C), *F. oxysporum* f. sp. *lycopersici* (FOXG_02147) and *F. graminearum* (FGRAMPH1_01G24387) retrieved from FungiDB (https://fungidb.org).

### 4.6 Generation of Plasmids and *C. graminicola* Strains

A ΔCgste3 deletion mutant and the complementation strain ΔCgste3::Cgste3 were generated to analyse whether the a‐pheromone receptor CgSte3 is involved in the sensing of MRE and diterpenoids. Assembly of the corresponding plasmids was conducted using the NEBuilder HiFi DNA Assembly Cloning Kit (New England Biolabs) according to the instruction manual. All plasmids were verified using DNA hydrolysis and sequencing using appropriate enzymes and primers. Oligonucleotides, strains and plasmids used are listed in Tables [Link], [Link], [Link].

As basis for homologous recombination in *C. graminicola* , the plasmid pCgste3_KO was generated by the assembly of three PCR fragments into pJet1.2 (Thermo Scientific). The fragments 5′ *Cgste3* (Ste3_P_fw//Ste3_P_rv; 1043 bp) and 3′ *Cgste3* (Ste3_T_fw//Ste3_T_rv; 1031 bp) were amplified from *C. graminicola* genomic DNA (gDNA). The *hph* cassette, mediating resistance to hygromycin B, was generated using the plasmid pRS‐hyg as template (hph‐f//hph‐r; 1417 bp) (Bloemendal et al. 2012).

For ΔCgste3 complementation, the plasmid pCgste3_nat was constructed. The 5′ and 3′ regions of *Cgste3* were amplified together with the *Cgste3* gene in a PCR using gDNA of *C. graminicola* as a template (Ste3_c_fw//Ste3_c_rv; 3215 bp). As a backbone for the assembly reaction, pJet_nat linearised with EcoRV (Nordzieke 2022), resulting in a resistance to nourseothricin‐dihydrogen sulphate in *C. graminicola* strains transformed with this plasmid.

Prior to transformations in *C. graminicola* , the plasmids pCgste3_KO and pCgste3_nat were linearised using the enzymes NaeI and ScaI, respectively. Oval conidia of CgM2 (transformation of pCgste3_KO) or ΔCgste3 (transformation of pCgste3_nat) served as the basis for the generation of protoplasts as described previously (Groth et al. 2021). After transformation, regenerating protoplasts were selected on medium containing hygromycin B (500 μg/mL, transformation of pCgste3_KO) or nourseothricin‐dihydrogen sulphate (70 μg/mL, transformation of pCgste3_nat). To obtain homokaryotic strains, single‐spore isolations were performed of antibiotic‐resistant and PCR‐verified primary transformants (Nordzieke 2022).

Single‐spore isolates of ΔCgste3 were verified by Southern blot analyses. Hydrolysis of gDNA was performed with the enzyme NaeI (Thermo Scientific). For the visualisation of successful deletion of the *Cgste3* gene via homologous recombination, the 3′ region of *Cgste3* was amplified in a PCR (Ste3_T_fw//Ste3_T_rv; 1031 bp) and used as a specific probe in the following hybridisation reaction (expected sizes: CgM2 2196 bp, ΔCgste3 7896 bp, Figure S5). Re‐integration of *Cgste3* into the ΔCgste3 deletion strain was tested via PCR using the primer pair Ste3_fw//Ste3_rv (1136 bp), verifying ΔCgste3::Cgste3 primary transformants and single‐spore isolates (Figure S6).

### 4.7 Analysis of *C. graminicola* Germination Patterns in Soil

For quantification of germination in soil, 1.875 mL spores solution (1.5 × 10^6^ mL^−1^) of either oval or falcate conidia was mixed in 5 mL (1) vermiculite or (2) soil‐vermiculite mixture containing Einheitserde Classic (Patzer Erden GmbH) and vermiculite (Vermiculite Palabora, grain size 2–3 mm, Isola Vermiculite GmbH) in a ratio of 4:1. After incubation (0 h, 6 h, 24 h, 48 h, 10 days), 2.5 mL water was added to wash out remaining spores. To assess the amount of remaining ungerminated spores, these were quantified in a Neubauer counting chamber in at least four technical replicates in three biological replicates.

Testing the effect of MRE on falcate conidia germination, 50 μL spore solution (10^6^ mL^−1^) was dropped on water agar (1 L contains 10 g agarose, 10 g serva agar, 2.1 g NaNO_3_). After evaporation of the liquid, 20 μL MRE was added on top of the spores. Germination rate was determined after 6 h and 17 h of incubation on water agar in three biological replicates.

### 4.8 Root Infection Analyses

Root infection experiments were performed with *Zea mays* ‘Mikado’ (KWS SAAT SE). Unless otherwise stated, incubation of plants was performed in a PK 520 WLED plant chamber (Poly Klima Climatic Growth System) using a day/night cycle of 12 h at 26°C/12 h at 18°C. The strain CgM2::RH2B, expressing tdTomato‐labelled histone 2B (Nordzieke, Sanken, et al. 2019) was used for root infection experiments.

### 4.9 Monitoring of *Z. mays* Root Infection Symptoms

Seeds of *Z. mays* were surface sterilised in 10% sodium hypochlorite for 10 min and incubated for 5 days in rectangle Petri dishes (82.9923.422, Sarstedt) on wet blotting paper (BF2 580 × 600 mm, Sartorius). Incubation took place in darkness using 12 h temperature cycles (26°C/18°C). The developed roots of 5‐day‐old seedlings were soaked in solutions of either oval or falcate conidia of CgM2::RH2B (10^7^ mL^−1^) for 1 h at 100 rpm. As a mock control, roots were soaked in sterile demineralised water. For follow‐up microscopic examinations, the plants were incubated on wet blotting paper in a day/night cycle of 12 h 26°C/12 h 18°C. 2 mL of demineralised water was added on the blotting paper after 2 days to prevent drought stress induction. Developed symptoms on roots were microscopically examined 4 days post‐inoculation.

For the analysis of symptom development after 21 days, the root‐dipped plants were further grown in 40 g vermiculite (Vermiculite Palabora, grain size 2–3 mm, Isola Vermiculite GmbH). The pots were covered with disposal plastic bags and sealed (Sarstedt) to increase humidity. After 21 days of incubation, the length and biomass of the above‐ground plants were determined, pictures of the symptom development were taken, and spreading into stem material was assessed with real‐time PCR.

### 4.10 Identification of *C. graminicola* In Planta via Real‐Time PCR

We developed a PCR‐based identification method to monitor the spread of *C. graminicola* from infected roots into plant stems. First, oligonucleotides (ITS_P4_fw and ITS_P9_rv) were designed to bind to *C. graminicola* ‐specific sequences of ITS1 and ITS2 flanking 5.8S rRNA. The primer pair amplifies a 339 bp fragment on a template of *C. graminicola* gDNA. Importantly, no amplicons were generated using gDNA from fungi (*Sordaria macrospora*, *V. dahliae*, *Verticillium longisporum*, *Aspergillus fumigatus* and *Aspergillus nidulans*) and maize as template in verification PCRs (Figure S10a,b).

To obtain template DNA, stems from infected plants or mock treatments were surface sterilised for 2 min in 10% sodium hypochlorite solution. From the lower part, 0.5 cm of stem was discarded. The following 2 cm of the plant stems were frozen in liquid nitrogen and stored at −80°C (Figure S10b). The collected stem samples were ground in liquid nitrogen to a fine powder. 500 μL extraction buffer (0.2 M Tris‐HCl pH 8.5, 25 mM EDTA, 0.5% SDS, 0.25 M NaCl) was added to each sample. At this step, the plasmid peGFP‐Cgatg8_gen (1 μL of 0.05 ng/μL) was added as an external standard for DNA losses during gDNA extraction. Further extraction was achieved with 8 M potassium acetate and chloroform. DNA was precipitated with ice‐cold isopropanol at −20°C for 2 h to overnight. The resulting pellet was resuspended in 50 μL demineralised water plus 2 μL 1 mg/mL RNase. As template for real‐time PCR, 300 ng of gDNA was used as a standard in all samples. Due to these high gDNA amounts, the calculated PCR efficiency (LinReg, https://www.gear‐genomics.com; Rausch et al. 2020, Rausch et al. 2019; Untergasser et al. 2021) showed elevated values (157.4%). Because the ratio of fungal versus plant gDNA in those samples is very low, we are not able to reduce the amount of template further without abolishing amplification. However, we never observed amplification in mock plant samples and thus we consider our approach as robust. The external standard plasmid peGFP‐Cgatg8_gen served as a template for amplification with the oligonucleotide pair GFP‐f and GFP‐r. The MESA GREEN qPCR MasterMix Plus for SYBR Assay (Eurogentec) together with a CFX Connect Real‐Time PCR Detection System (Bio‐Rad Laboratories) was used for detection. In all experiments, three technical replicates were used to generate one biological replicate. Follow‐up DNA sequencing and BLAST analyses verified the identity of the obtained fragment as originating from *C. graminicola* (Figure S10c).

### 4.11 Microscopy

Light (differential interference contrast [DIC]) and fluorescence microscopy was performed with the Axiolmager M1 microscope (Zeiss). The Photometrix coolSNAP HQ camera (Roper Scientific, Photometrics) was used to capture images, and processing was performed with the ZEN software (v. 2.3; Zeiss). Visualisation of expressed red‐fluorescent CgM2::RH2B was achieved by a 49,005 Chroma filter set (exciter ET545/30×, emitter ET620/60 and beam splitter T570LP). At least three biological replicates were performed for each experiment.

### 4.12 Statistics

The *t* test for unequal variances was used for all experiments in this study (Ruxton 2006).

### 4.13 Artificial Intelligence

Artificial intelligence tool Chat AI provided by the GWDG at the University of Göttingen was used to improve English spelling and grammar.

## Author Contributions

**Anina Y. Rudolph:** methods, investigation, writing of original draft, revision of text. **Carolin Schunke:** methods, investigation, revision of text. **Christoph Sasse:** investigation, revision of text. **Luis Antelo:** investigation, revision of text. **Jennifer Gerke:** investigation, revision of text. **Gerhard H. Braus:** funding, revision of text. **Stefanie Pöggeler:** revision of text, resources. **Daniela E. Nordzieke:** investigation, resources, writing of original draf, revision of text, funding.

## Conflicts of Interest

The authors declare no conflicts of interest.

## Supporting information


**Figure S1:** Representative chromatogram of HPLC/MS analysis of maize root exudate (MRE) samples. (A–D) non‐attracting samples. (A) Demineralised water, (B) chloroform phase of demineralised water after chloroform/methanol extraction, (C) aqueous phase of demineralised water after chloroform/methanol extraction, (D) aqueous phase of MRE after chloroform/methanol extraction. (E–H) Attracting samples. (E) MRE, (F) boiled MRE, (G) chloroform phase of MRE after chloroform/methanol extraction, (H) MRE of plants co‐incubated with oval conidia of CgM2.

**Figure S2:** UV and MS2 spectra of compound 1, eluting at 12 min from HPLC (EIC m/z 305.1748). (a) UV/Vis spectra were recorded with a Thermo ScientificTm DionexTm Ultim ateTm 3000 Diode Array Detector. (b) MS2 data were recorded at a HCD energy of 35 eV.

**Figure S3:** Fractionation experiments of maize root exudate (MRE). (a) Different fractions of MRE obtained after first fraction using HPLC analysis. (b) Chemotropic growth of *Colletotrichum graminicola* germlings derived from oval conidia to different MRE fractions, *n* = 1. Fractions F4‐F6 and F12‐F13 were used for further purification.

**Figure S4:** Identification and in silico analysis of the a‐pheromone receptor CgSte3. (a) Protein structure prediction of GLRG_03765 with AlphaFold Protein Structure Database showed the typical structure of a G‐protein coupled receptor with 6 transmembrane Domains and a long C‐terminal extension (Zhang et al. 2015). (b) Protein alignment was performed with ClustalOmega (Madeira et al. 2024, alignment style: Nightingale, colour scheme: clustal2) with sequences of homologous proteins of *Saccharomyces cerevisiae* (YKL178C), *Fusarium oxysporum* f. sp. *lycopersici* (FOXG_02147) and *Fusarium graminearum* (FGRAMPH1_01G24387) retrieved from FungiDB (Alvarez‐Jarreta et al. 2024).

**Figure S5:** Generation of a *Cgste3* deletion strain in *Colletotrichum graminicola* . (a) Strategy for ΔCgste3 generation. Genomic loci of *Cgste3* in CgM2 wildtype and deletion strain. Primer binding sites for the generation of the deletion construct are indicated, arrows indicate the amplification direction, red arrows indicate the oligonucleotides used for the generation of a *Cgste3*‐specific probe for Southern blot analysis. Recognition sites for NaeI and the expected band sizes are depicted in blue. (b) Verification of homologous integration of a *hph*‐resistance cassette into the *Cgste3* locus via Southern blot hybridisation. Expected band sizes after hydrolysis of gDNA with NaeI are indicated with black (CgM2: 2196 bp) and white arrowheads (ΔCgste3: 7896 bp). Strains used for phenotypic characterisation are written in bold letters, the strain used for complementation is additionally indicated with an asterisk.

**Figure S6:** Verification of ΔCgste3::Cgste3. Amplification with the oligonucleotides Ste3_fw and Ste3_rv with an expected band size of 1136 bp (black arrowhead).

**Figure S7:** Germination of *Colletotrichum graminicola* falcate conidia in different soil substrates. Oval or falcate conidia of the strain CgM2::RH2B expressing tdTomato‐Histone2B were mixed in 5 mL of (1) vermiculite or (2) soil‐vermiculite mixture containing of Einheitserde Classic (Patzer Erden GmbH) and vermiculite (Vermiculite Palabora, grain size 2–3 mm, Isola Vermiculite GmbH) in a ratio 4:1. After incubation for 24 h, microscopic pictures were taken.

**Figure S8:** Microscopic assessment of *Colletotrichum graminicola* colonising and infecting roots of *Zea mays* . Roots of 5‐day‐old maize plants were soaked in suspensions of oval (oc) or falcate (fc) conidia (10^7^ mL^−1^) and incubated on wet blotting paper for 4 day. RH = runner hypha, MS = microsclerotium, HP = hyphopodium, scale bar = 50 μm.

**Figure S9:** Symptom development after dipping root infection of *Zea mays* with *Colletotrichum graminicola* . Roots of 5‐day‐old plants were dipped in conidial suspensions. Subsequently, the plants were grown in vermiculite for 21 days. Quantification of length and biomass of the above‐ground plant parts. Error bars represent SD calculated from *n* ≥ 14 experiments, ns = not significant.

**Figure S10:** Establishment of the real‐time PCR to quantify plants with spreading of *Colletotrichum graminicola* in stem tissue. (a, b) The primer ITS_P4_fw and ITS_P9_rv are designed to specifically bind CgM2 but no other fungi shown for *Sordaria macrospora* (Sm), *Aspergillus fumigatus* (Af), *Aspergillus nidulans* (An), *Verticillium longisporum* (Vl) and *Verticillium dahliae* (Vd) (a) or gDNA extracted from maize stems (b). bp = base pairs. (c) Schematic depiction of sample collection. 0.5 cm above the kernel were discarded and gDNA extraction was performed from 2 cm stem above. (d) Alignment of CgM2 sequences using NCBI/BLAST. The expected CgM2 sequence spanning ITS1, the 5.8S rRNA encoding region and ITS2 (CgM2) was aligned with the sequence obtained of stems of root‐infected plants (dRI). (e) Melting curve of primer pair ITS_P4_fw and ITS_P9_rv. Upper curves show the performance when a gDNA sample is used in three technical replicates, the baseline curves the performance of no‐template control technical replicates.

**Table S1:** Oligonucleotides used in this study.

**Table S2:** mpp70155‐sup‐0012‐TableS2.docx. *Colletotrichum graminicola* strains used in this study.

**Table S3:** Plasmids used in this study.

**Table S4:** Real‐time PCR results obtained with stems of not infected (Mock) or root‐infected maize plants.

## Data Availability

The data that support the findings of this study are available from the corresponding author upon reasonable request.
