# Supplementary figures and images for: Maize Diterpenoid Sensing via the Ste3 A‐Pheromone Receptor Guide Oval Conidia of Colletotrichum graminicola to Host Roots
Source: Mol Plant Pathol. 2025 Sep 18;26(9):e70155. doi: 10.1111/mpp.70155 (PMC12445352; doi:10.1111/mpp.70155)

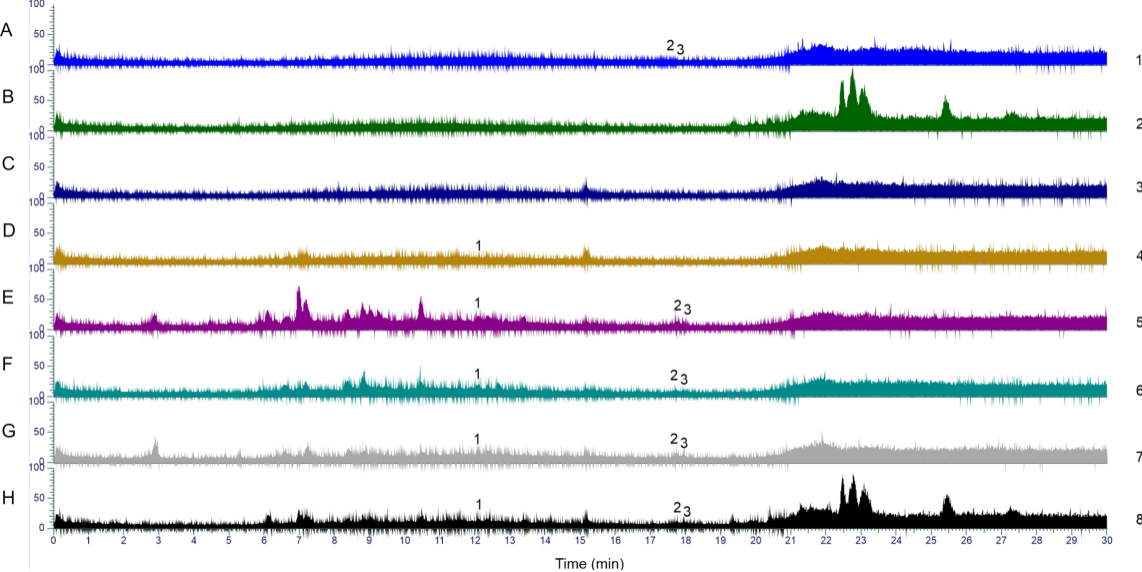

Supplement: Supplementary file 1 — Figure S1: Representative chromatogram of HPLC/MS analysis of maize root exudate (MRE) samples. (A–D) non‐attracting samples. (A) Demineralised water, (B) chloroform phase of demineralised water after chloroform/methanol extraction, (C) aqueous phase of demineralised water after chloroform/methanol extraction, (D) aqueous phase of MRE after chloroform/methanol extraction. (E–H) Attracting samples. (E) MRE, (F) boiled MRE, (G) chloroform phase of MRE after chloroform/methanol extraction, (H) MRE of plants co‐incubated with oval conidia of CgM2. [file MPP-26-e70155-s002.pdf]

(a)

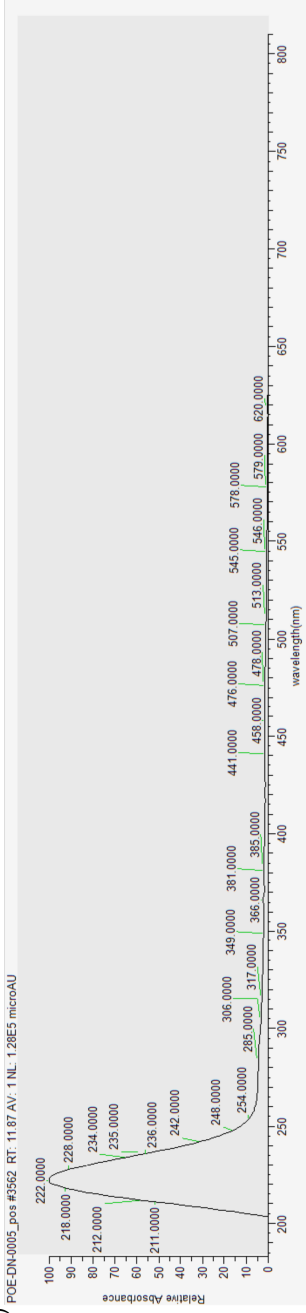

(b)

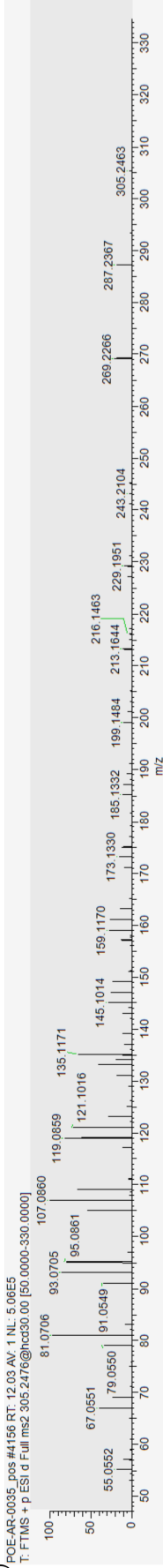

Supplement: Supplementary file 2 — Figure S2: UV and MS2 spectra of compound 1, eluting at 12 min from HPLC (EIC m/z 305.1748). (a) UV/Vis spectra were recorded with a Thermo ScientificTm DionexTm Ultim ateTm 3000 Diode Array Detector. (b) MS2 data were recorded at a HCD energy of 35 eV. [file MPP-26-e70155-s006.pdf]

(a)

a

MRE fractionation

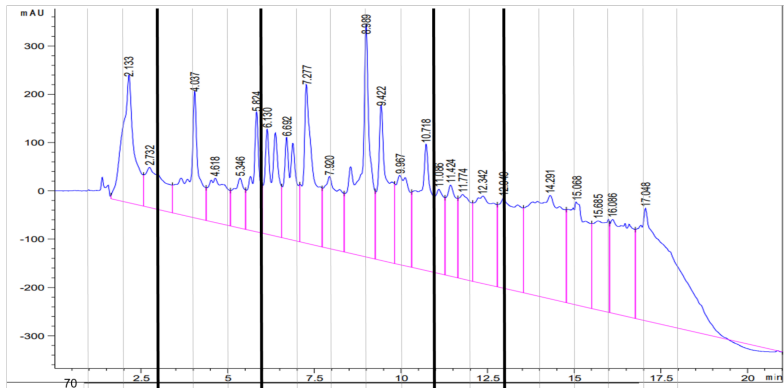

(b)

Chemotropic growth  
of MRE fractions

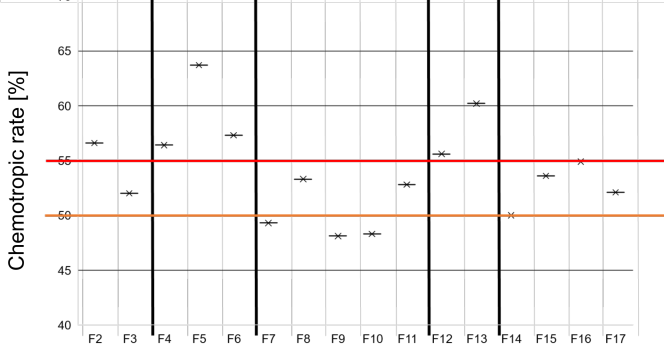

Supplement: Supplementary file 3 — Figure S3: Fractionation experiments of maize root exudate (MRE). (a) Different fractions of MRE obtained after first fraction using HPLC analysis. (b) Chemotropic growth of Colletotrichum graminicola germlings derived from oval conidia to different MRE fractions, n = 1. Fractions F4‐F6 and F12‐F13 were used for further purification. [file MPP-26-e70155-s014.pdf]

(a)

NaeI

NaeI

2,196 bp

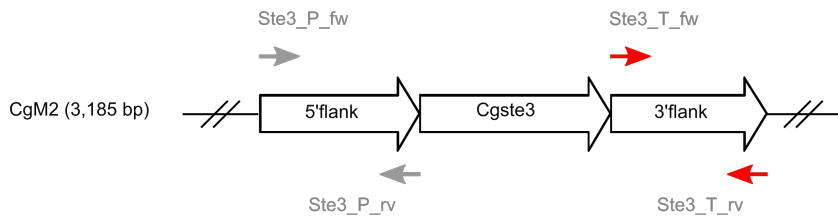

NaeI

NaeI

7,698 bp

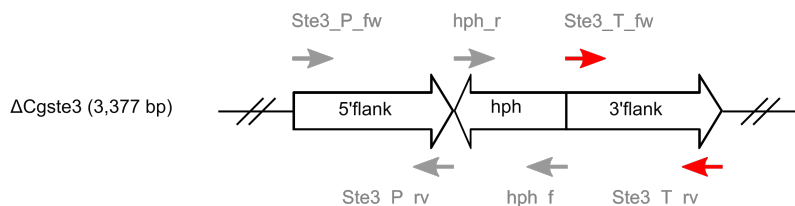

(b)

ΔCgste3

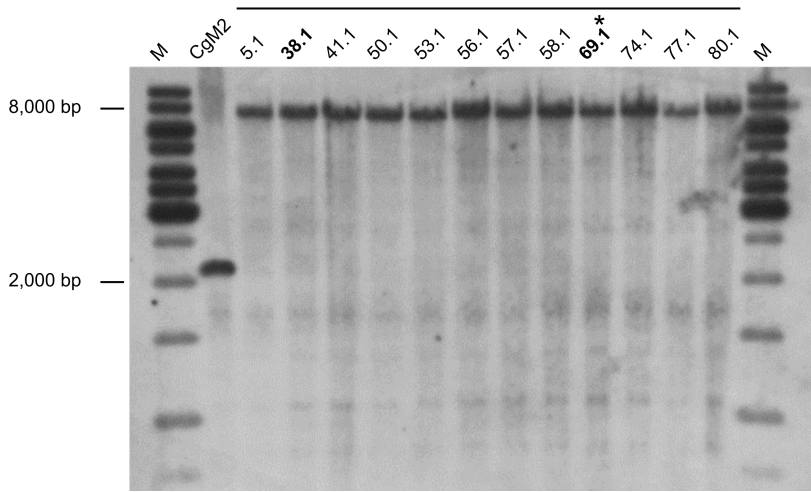

Supplement: Supplementary file 5 — Figure S5: Generation of a Cgste3 deletion strain in Colletotrichum graminicola . (a) Strategy for ΔCgste3 generation. Genomic loci of Cgste3 in CgM2 wildtype and deletion strain. Primer binding sites for the generation of the deletion construct are indicated, arrows indicate the amplification direction, red arrows indicate the oligonucleotides used for the generation of a Cgste3‐specific probe for Southern blot analysis. Recognition sites for NaeI and the expected band sizes are depicted in blue. (b) Verification of homologous integration of a hph‐resistance cassette into the Cgste3 locus via Southern blot hybridisation. Expected band sizes after hydrolysis of gDNA with NaeI are indicated with black (CgM2: 2196 bp) and white arrowheads (ΔCgste3: 7896 bp). Strains used for phenotypic characterisation are written in bold letters, the strain used for complementation is additionally indicated with an asterisk. [file MPP-26-e70155-s012.pdf]

$\Delta$ Cgste3::Cgste3

CgM2

1.1

5.2

6.1

6.2

H<sub>2</sub>O

1,000 bp —

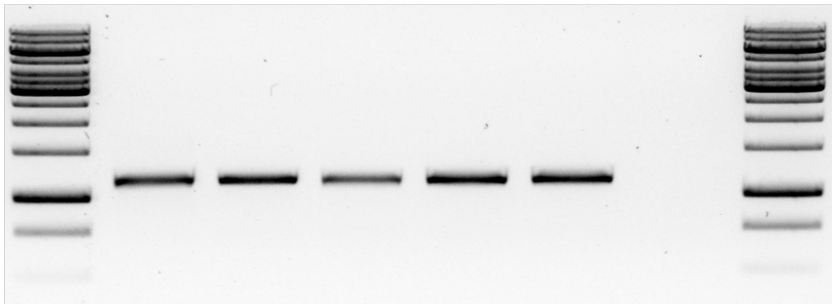

Supplement: Supplementary file 6 — Figure S6: Verification of ΔCgste3::Cgste3. Amplification with the oligonucleotides Ste3_fw and Ste3_rv with an expected band size of 1136 bp (black arrowhead). [file MPP-26-e70155-s003.pdf]

Vermiculite

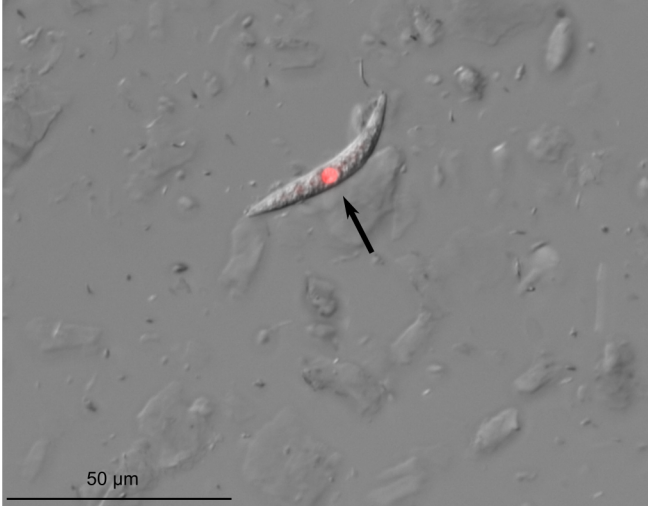

4+1 soil(l)-vermiculite mixture

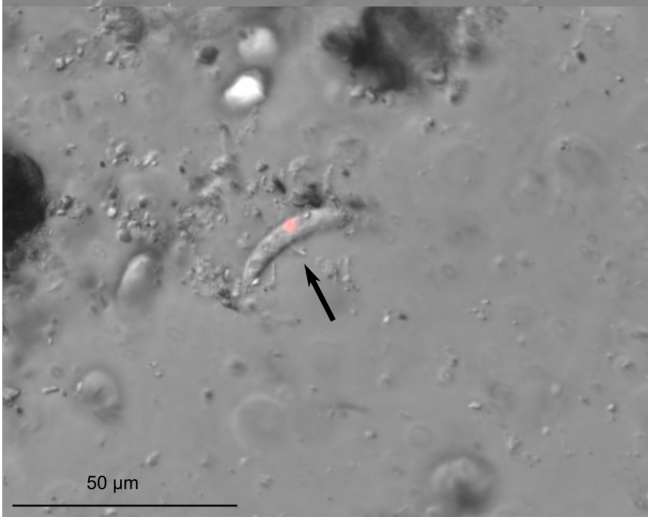

Supplement: Supplementary file 7 — Figure S7: Germination of Colletotrichum graminicola falcate conidia in different soil substrates. Oval or falcate conidia of the strain CgM2::RH2B expressing tdTomato‐Histone2B were mixed in 5 mL of (1) vermiculite or (2) soil‐vermiculite mixture containing of Einheitserde Classic (Patzer Erden GmbH) and vermiculite (Vermiculite Palabora, grain size 2–3 mm, Isola Vermiculite GmbH) in a ratio 4:1. After incubation for 24 h, microscopic pictures were taken. [file MPP-26-e70155-s009.pdf]

oc

fc

runner hyphae

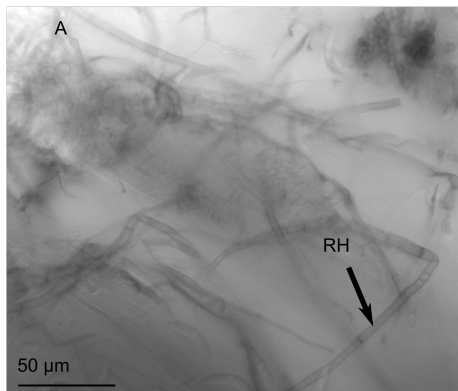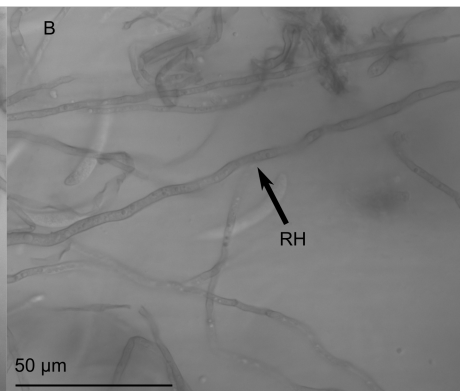

microsclerotia

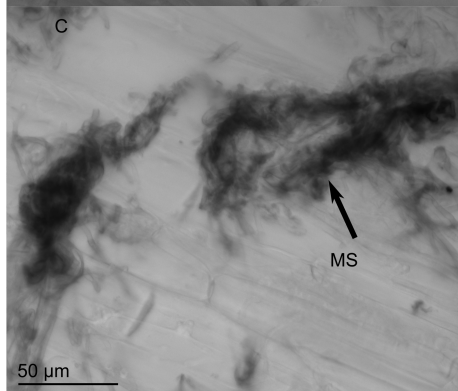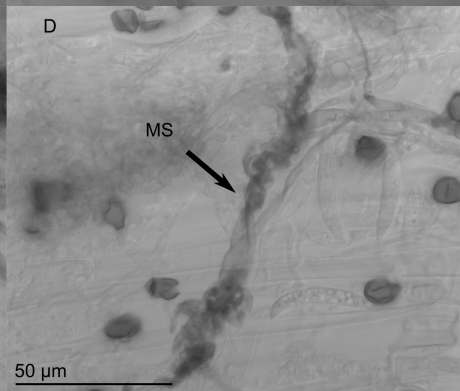

hyphopodia

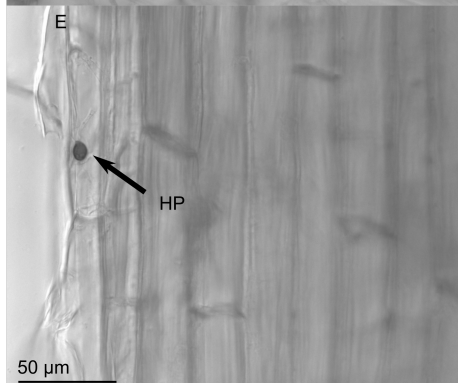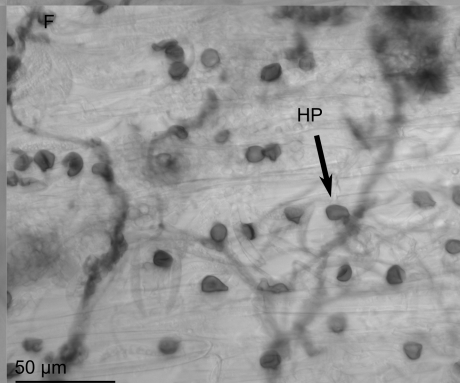

Supplement: Supplementary file 8 — Figure S8: Microscopic assessment of Colletotrichum graminicola colonising and infecting roots of Zea mays . Roots of 5‐day‐old maize plants were soaked in suspensions of oval (oc) or falcate (fc) conidia (107 mL−1) and incubated on wet blotting paper for 4 day. RH = runner hypha, MS = microsclerotium, HP = hyphopodium, scale bar = 50 μm. [file MPP-26-e70155-s005.pdf]

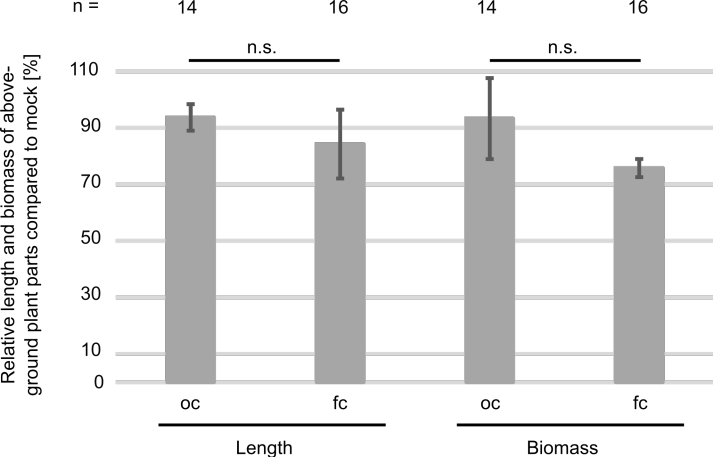

Supplement: Supplementary file 9 — Figure S9: Symptom development after dipping root infection of Zea mays with Colletotrichum graminicola . Roots of 5‐day‐old plants were dipped in conidial suspensions. Subsequently, the plants were grown in vermiculite for 21 days. Quantification of length and biomass of the above‐ground plant parts. Error bars represent SD calculated from n ≥ 14 experiments, ns = not significant. [file MPP-26-e70155-s001.pdf]
