# Supplementary material for: Maize Diterpenoid Sensing via the Ste3 A‐Pheromone Receptor Guide Oval Conidia of Colletotrichum graminicola to Host Roots
Source: Mol Plant Pathol. 2025 Sep 18;26(9):e70155. doi: 10.1111/mpp.70155 (PMC12445352; doi:10.1111/mpp.70155)
Supplement: Supplementary file 4 — Figure S4: Identification and in silico analysis of the a‐pheromone receptor CgSte3. (a) Protein structure prediction of GLRG_03765 with AlphaFold Protein Structure Database showed the typical structure of a G‐protein coupled receptor with 6 transmembrane Domains and a long C‐terminal extension (Zhang et al. 2015). (b) Protein alignment was performed with ClustalOmega (Madeira et al. 2024, alignment style: Nightingale, colour scheme: clustal2) with sequences of homologous proteins of Saccharomyces cerevisiae (YKL178C), Fusarium oxysporum f. sp. lycopersici (FOXG_02147) and Fusarium graminearum (FGRAMPH1_01G24387) retrieved from FungiDB (Alvarez‐Jarreta et al. 2024). [file MPP-26-e70155-s013.pdf]

(a)

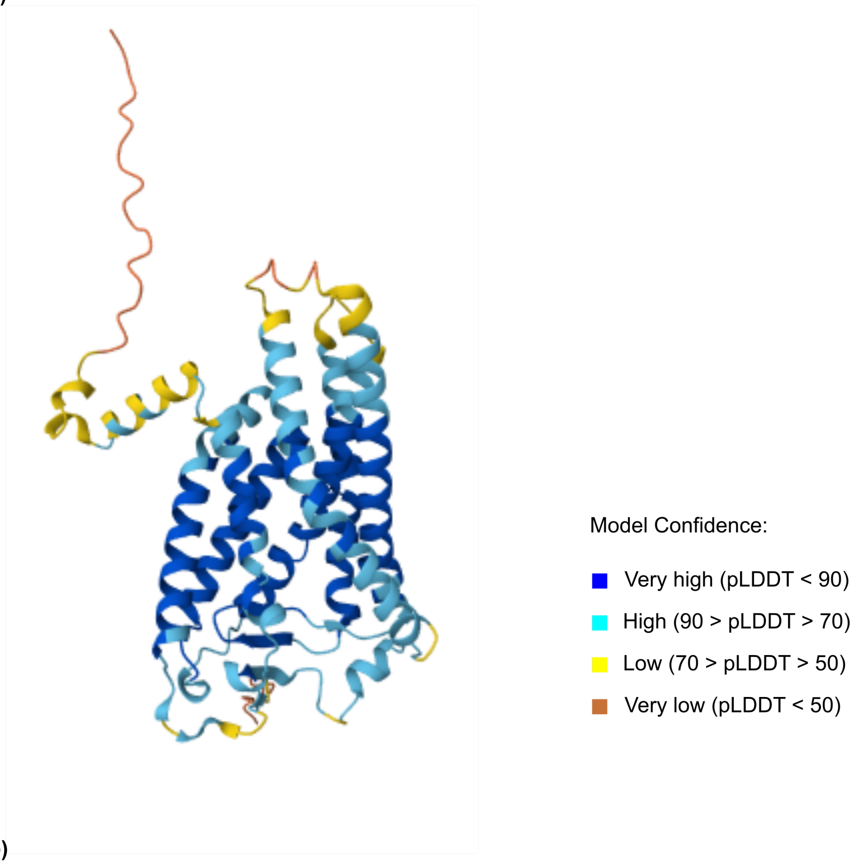
Model Confidence:
Very high (pLDDT < 90)
High (90 > pLDDT > 70)
Low (70 > pLDDT > 50)
Very low (pLDDT < 50)

(b)

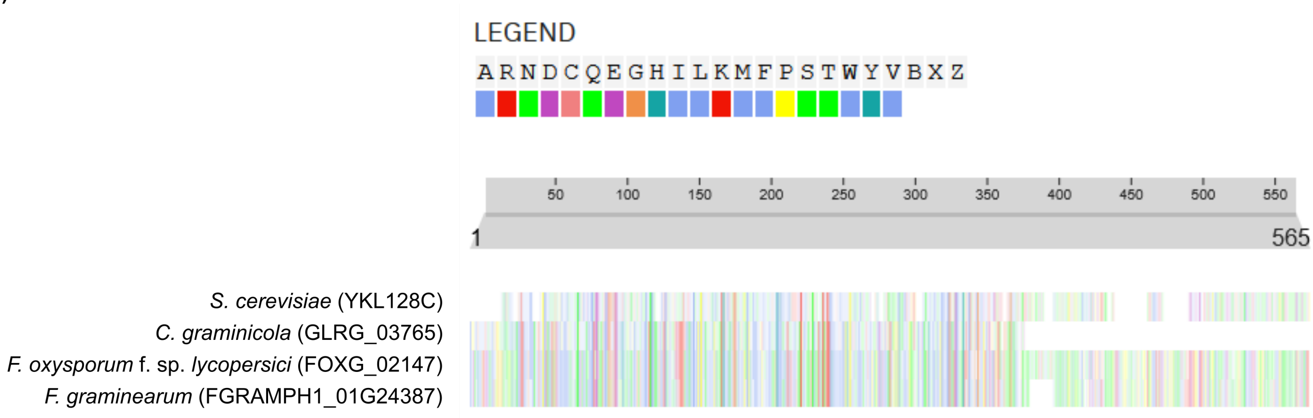
LEGEND
A R N D C Q E G H I L K M F P S T W Y V B X Z
50 100 150 200 250 300 350 400 450 500 550
1
565
S. cerevisiae (YKL128C)
C. graminicola (GLRG_03765)
F. oxysporum f. sp. lycopersici (FOXG_02147)
F. graminearum (FGRAMPH1_01G24387)
