# Supplementary material for: Maize Diterpenoid Sensing via the Ste3 A‐Pheromone Receptor Guide Oval Conidia of Colletotrichum graminicola to Host Roots
Source: Mol Plant Pathol. 2025 Sep 18;26(9):e70155. doi: 10.1111/mpp.70155 (PMC12445352; doi:10.1111/mpp.70155)
Supplement: Supplementary file 10 — Figure S10: Establishment of the real‐time PCR to quantify plants with spreading of Colletotrichum graminicola in stem tissue. (a, b) The primer ITS_P4_fw and ITS_P9_rv are designed to specifically bind CgM2 but no other fungi shown for Sordaria macrospora (Sm), Aspergillus fumigatus (Af), Aspergillus nidulans (An), Verticillium longisporum (Vl) and Verticillium dahliae (Vd) (a) or gDNA extracted from maize stems (b). bp = base pairs. (c) Schematic depiction of sample collection. 0.5 cm above the kernel were discarded and gDNA extraction was performed from 2 cm stem above. (d) Alignment of CgM2 sequences using NCBI/BLAST. The expected CgM2 sequence spanning ITS1, the 5.8S rRNA encoding region and ITS2 (CgM2) was aligned with the sequence obtained of stems of root‐infected plants (dRI). (e) Melting curve of primer pair ITS_P4_fw and ITS_P9_rv. Upper curves show the performance when a gDNA sample is used in three technical replicates, the baseline curves the performance of no‐template control technical replicates. [file MPP-26-e70155-s008.pdf]

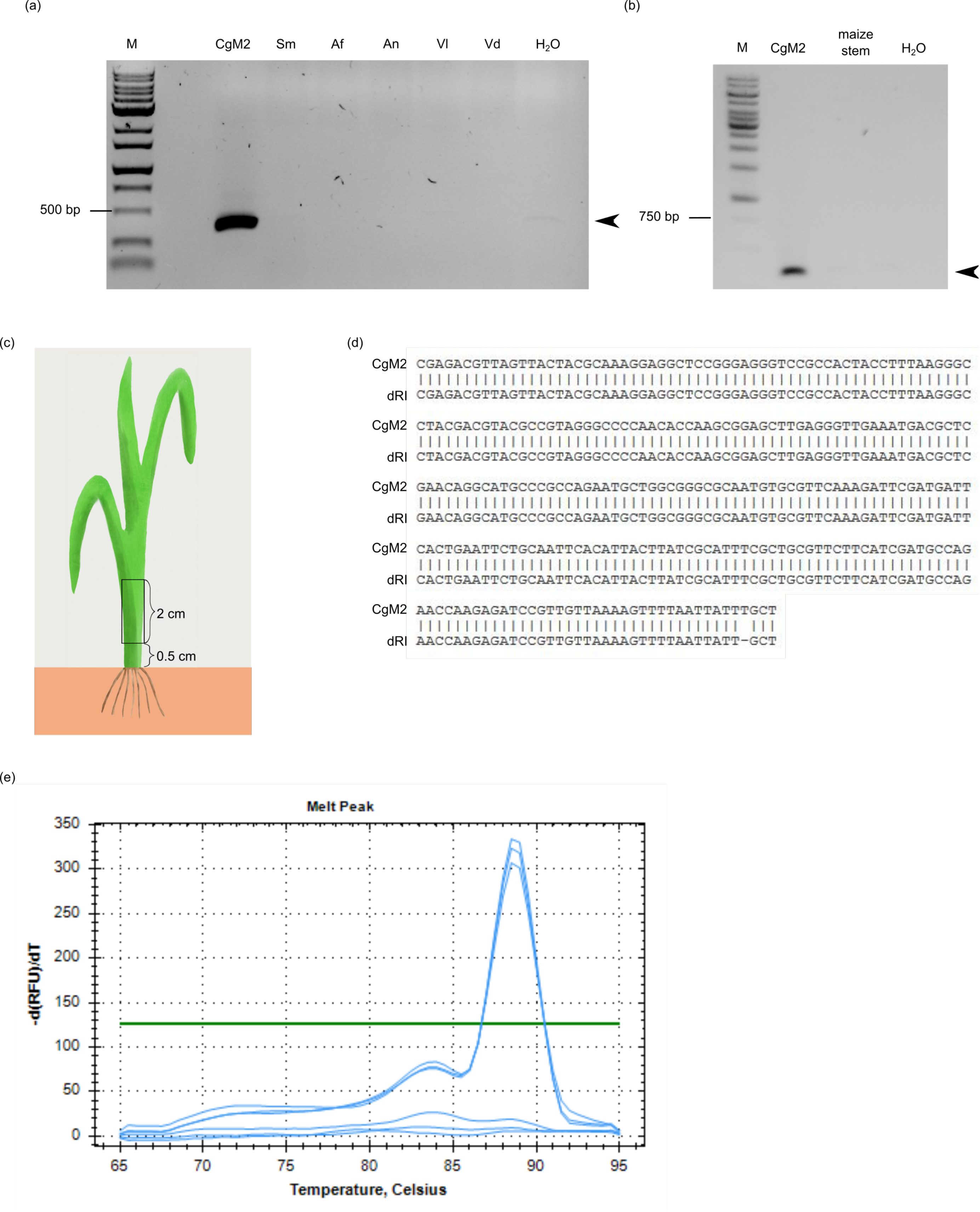

(a)
M
CgM2
Sm
Af
An
Vl
Vd
H2O
500 bp
(b)
M
CgM2
maize stem
H2O
750 bp
(c)
2 cm
0.5 cm
(d)
CgM2 CGAGACGTTAGTTACTACGCAAAGGAGGCTCCGGGAGGGTCCGCCACTACCTTTAAGGGC
dRI CGAGACGTTAGTTACTACGCAAAGGAGGCTCCGGGAGGGTCCGCCACTACCTTTAAGGGC
CgM2 CTACGACGTACGCCGTAGGGCCCCAACACCAAGCGGAGCTTGAGGGTTGAAATGACGCTC
dRI CTACGACGTACGCCGTAGGGCCCCAACACCAAGCGGAGCTTGAGGGTTGAAATGACGCTC
CgM2 GAACAGGCATGCCCGCCAGAATGCTGGCGGGCGCAATGTGCGTTCAAAGATTCGATGATT
dRI GAACAGGCATGCCCGCCAGAATGCTGGCGGGCGCAATGTGCGTTCAAAGATTCGATGATT
CgM2 CACTGAATTCTGCAATTCACATTACTTATCGCATTTCGCTGCGTTCTTCATCGATGCCAG
dRI CACTGAATTCTGCAATTCACATTACTTATCGCATTTCGCTGCGTTCTTCATCGATGCCAG
CgM2 AACCAAGAGATCCGTTGTTAAAAGTTTTAATTATTTGCT
dRI AACCAAGAGATCCGTTGTTAAAAGTTTTAATTATT-GCT
(e)
Melt Peak
-d(RFU)/dT
Temperature, Celsius
0
50
100
150
200
250
300
350
65
70
75
80
85
90
95
