# Supplementary material for: Maize Diterpenoid Sensing via the Ste3 A‐Pheromone Receptor Guide Oval Conidia of Colletotrichum graminicola to Host Roots
Source: Mol Plant Pathol. 2025 Sep 18;26(9):e70155. doi: 10.1111/mpp.70155 (PMC12445352; doi:10.1111/mpp.70155)
Supplement: Supplementary file 11 — Table S1: Oligonucleotides used in this study. [file MPP-26-e70155-s010.docx]

**Table S1** **Oligonucleotides used in this study.**

| Oligonucleotide | Sequence (5` to 3`) |
| --- | --- |
| ITS_P4_fw | GCCGGAGGATAACCAAACTCTG |
| ITS_P9_rv | GATCCCGATGCGAGACGTTAG |
| GFP-f | ATGGTGAGCAAGGGCGAGGAGC |
| GFP-r | CTTGTACAGCTCGTCCATGCCGAGAGTG |
| hph-f | gttaactgatattgaaggagcatttttgg |
| hph-r | gttaactggttcccggtcggcatctactc |
| ste3_P_fw | GTAACGCCAGGGTTTTCCCAGTCACGACG CAATTGTACCCCTCTTCCCGTAC |
| ste3_P_rv | GAGTAGATGCCGACCGGGAACCAGTTAAC CTAAGTAAGTGTTCGAAACGACGC |
| ste3_T_fw | CCAAAAATGCTCCTTCAATATCAGTTAAC CTCGCTTTATCCCAGAACGTTG |
| ste3_T_rv | GCGGATAACAATTTCACACAGGAAACAGC GGACGAACCGACATGAATTTATACG |
| ste3_c_fw | CTTCCGGATGGCGAT CAATTGTACCCCTCTTCCCGTAC |
| ste3_c_rv | CCCTGCCCCTGAGAT GGACGAACCGACATGAATTTATACG |
| Ste3_fw | CACAGTACACCAACGCCTATCTC |
| Ste3_rv | CTACTCTGTCTCTACCTGCAGC |

Overhangs for the assembly reactions are indicated in red.
