# Supplementary material for: Maize Diterpenoid Sensing via the Ste3 A‐Pheromone Receptor Guide Oval Conidia of Colletotrichum graminicola to Host Roots
Source: Mol Plant Pathol. 2025 Sep 18;26(9):e70155. doi: 10.1111/mpp.70155 (PMC12445352; doi:10.1111/mpp.70155)
Supplement: Supplementary file 12 — Table S2: mpp70155‐sup‐0012‐TableS2.docx. Colletotrichum graminicola strains used in this study. [file MPP-26-e70155-s004.docx]

**Table S2** ***Colletotrichum graminicola strains used in this study.***

| Strain | Genotype | Reference |
| --- | --- | --- |
| CgM2 (M1.001) | *C. graminicola* wild-type (wt) | (Forgey *et al.*, 1978) |
| CgM2::arp1-tagRFP-T | ectopic integration of pCgarp1-TagRFP-T in CgM2; *gen^R^*, ssi, *Cgarp1P::Cgarp1::TagRFP-T::TtrpC* | (Groth *et al.*, 2021) |
| CgM2::RH2B | ectopic integration of Pgpd::hh2b::tdTomato::TtrpC in CgM2; *hyg^R^*, ssi, *CgM2::rh2b* | (Nordzieke *et al.*, 2019) |
| ΔCgste3 | Homologous replacement of *Cgste3* in CgM2, ssi, *hyg*^R^, *Cgste3*::*hph*, | this study |
| ΔCgste3::Cgste3 | Ectopic integration of pCgste3_nat in ∆Cgste3, *nat*^R^, ssi; ∆*Cgste3*::*Cgste3* | this study |

*gen^R^*: resistant to geniticin; *nat^R^*: resistant to nourseothricin; *hyg^R^*: hygromycin resistant; ssi: single spore isolate

References

Forgey, W., Blanco, M. and Loegering, W. (1978) "Differences in pathological capabilities and host specificity of *Colletotrichum graminicola* on *Zea mays*." *Plant Disease Reporter,* 62**,** 573-576.

Groth, A., Schunke, C., Reschka, E. J., Pöggeler, S. and Nordzieke, D. E. (2021) "Tracking fungal growth: Establishment of Arp1 as a marker for polarity establishment and active hyphal growth in filamentous ascomycetes." *Journal of Fungi,* 7**,** 580.

Nordzieke, D. E., Sanken, A., Antelo, L., Raschke, A., Deising, H. B. and Pöggeler, S. (2019) "Specialized infection strategies of falcate and oval conidia of *Colletotrichum graminicola*." *Fungal Genetics Biology,* 133**,** 103276.
