# Supplementary material for: Maize Diterpenoid Sensing via the Ste3 A‐Pheromone Receptor Guide Oval Conidia of Colletotrichum graminicola to Host Roots
Source: Mol Plant Pathol. 2025 Sep 18;26(9):e70155. doi: 10.1111/mpp.70155 (PMC12445352; doi:10.1111/mpp.70155)
Supplement: Supplementary file 13 — Table S3: Plasmids used in this study. [file MPP-26-e70155-s011.docx]

**Table S3** **Plasmids used in this study.**

| Plasmid | Features | Reference |
| --- | --- | --- |
| peGFP‐Cgatg8_gen | 5‘ Cgatg8::eGFP::Cgatg8::3’Cgatg8, genR, ampR | (Nordzieke, 2022) |
| pCgste3_KO | *5‘ Cgste3::hph::3’ Cgste3, hyg^R^, amp*^R^ | this study |
| pCgste3_nat | *5‘ Cgste3::Cgste3::3‘ Cgste3, nat^R^, amp^R^* | this study |
| pJet1.2 | *amp^R^* | ThermoFisher Scientific |
| pJet_nat | *nat^R^, amp^R^* | (Nordzieke, 2022) |

*amp^R^*: ampicillin resistant; *nat^R^*: resistant to nourseothricin; *URA3*: encodes for Orotidine-5'-phosphate (OMP) decarboxylase

References

Nordzieke, D. E. (2022) "Hyphal Fusions Enable Efficient Nutrient Distribution in *Colletotrichum graminicola* Conidiation and Symptom Development on Maize." *Microorganisms,* 10**,** 1146.
