# Supplementary material for: Maize Diterpenoid Sensing via the Ste3 A‐Pheromone Receptor Guide Oval Conidia of Colletotrichum graminicola to Host Roots
Source: Mol Plant Pathol. 2025 Sep 18;26(9):e70155. doi: 10.1111/mpp.70155 (PMC12445352; doi:10.1111/mpp.70155)
Supplement: Supplementary file 14 — Table S4: Real‐time PCR results obtained with stems of not infected (Mock) or root‐infected maize plants. [file MPP-26-e70155-s007.docx]

**Table S4** **Real time PCR results obtained with stems of not infected (Mock) or root-infected maize plants.**

| Sample | relative Expression | Expression SD | Expression SEM |
| --- | --- | --- | --- |
| Mock_1 | 0 | 0 | 0 |
| Mock_2 | 0 | 0 | 0 |
| Mock_3 | 0 | 0 | 0 |
| Mock_4 | 0 | 0 | 0 |
| Mock_5 | 0 | 0 | 0 |
| Mock_6 | 0 | 0 | 0 |
| Mock_7 | 0 | 0 | 0 |
| Mock_8 | 0 | 0 | 0 |
| oc_1 | 0 | 0 | 0 |
| oc_2 | 0 | 0 | 0 |
| oc_3 | 13,30 | 3,40 | 1,97 |
| oc_4 | 0,02 | 0,01 | 0,00 |
| oc_5 | 0 | 0 | 0 |
| oc_6 | 0 | 0 | 0 |
| oc_7 | 8,06 | 10,77 | 6,22 |
| oc_8 | 0,13 | 0,04 | 0,03 |
| fc_1 | 0 | 0 | 0 |
| fc_2 | 0 | 0 | 0 |
| fc_3 | 0 | 0 | 0 |
| fc_4 | 0 | 0 | 0 |
| fc_5 | 0 | 0 | 0 |
| fc_6 | 0 | 0 | 0 |
| fc_7 | 0 | 0 | 0 |
| fc_8 | 0 | 0 | 0 |
